# Transition-Metal Isocorroles as Singlet Oxygen Sensitizers

**DOI:** 10.1021/acs.inorgchem.3c00782

**Published:** 2023-05-04

**Authors:** Simon Larsen, Joseph A. Adewuyi, Gaël Ung, Abhik Ghosh

**Affiliations:** † Department of Chemistry, University of Tromsø, N-9037 Tromsø, Norway; ‡ Department of Chemistry, University of Connecticut, 55 N. Eagleville Rd, Storrs, Connecticut 06269, United States

## Abstract

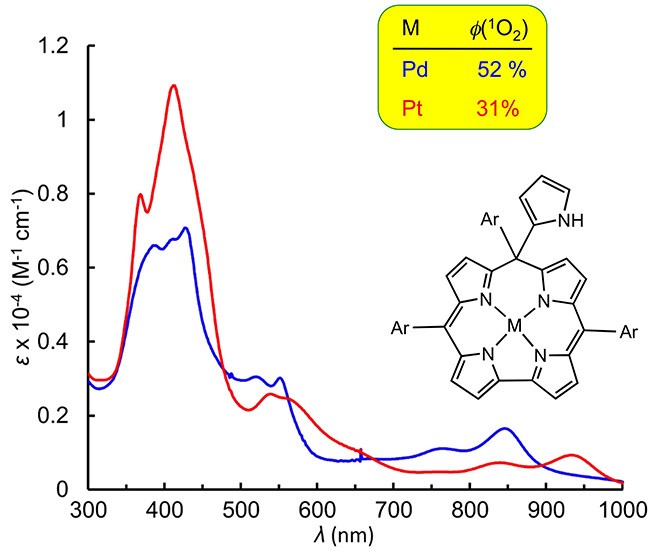

Building on a highly efficient synthesis of pyrrole-appended isocorroles, we have worked out conditions for manganese, palladium, and platinum insertion into free-base 5/10-(2-pyrrolyl)-5,10,15-tris(4-methylphenyl)isocorrole, H_2_[5/10-(2-py)T*p*MePiC]. Platinum insertion proved exceedingly challenging but was finally accomplished with *cis*-Pt(PhCN)_2_Cl_2_. All the complexes proved weakly phosphorescent in the near-infrared under ambient conditions, with a maximum phosphorescence quantum yield of 0.1% observed for Pd[5-(2-py)T*p*MePiC]. The emission maximum was found to exhibit a strong metal ion dependence for the 5-regioisomeric complexes but not for the 10-regioisomers. Despite the low phosphorescence quantum yields, all the complexes were found to sensitize singlet oxygen formation with moderate to good efficiency, with singlet oxygen quantum yields ranging over 21–52%. With significant absorption in the near-infrared and good singlet oxygen-sensitizing ability, metalloisocorroles deserve examination as photosensitizers in the photodynamic therapy of cancer and other diseases.

## Abstract 2

The Mn(II), Pd(II), and Pt(II) derivatives of pyrrole-appended isocorroles exhibit exceedingly weak NIR phosphorescence but moderate to good singlet oxygen quantum yields of 21−52%.

## Introduction

Isoporphyrin, first reported by Dolphin over 50 years ago,^1^ and related porphyrinoid macrocycles^2,3^ such as phlorin^4,5^ and isocorrole^6,7^ are currently enjoying a renaissance.^8^ The reasons are manifold. Isoporphyrinoids are fascinating hybrid ligands:^9,10^ isocorrole, for example, resembles a corrole in terms of its constricted central cavity, but it resembles porphyrin in terms of its dianionic character as a ligand.^11,12^ Second, depending on the substituents at the saturated *meso* carbon, isoporphyrinoids are of considerable theoretical interest on account of their homoaromatic or homoantiaromatic character.^13^ Third, isoporphyrins and isocorroles exhibit strong near-infrared (NIR) absorption and may prove useful in bioimaging and as sensitizers in photodynamic therapy.^14^ Finally, isoporphyrinoids geminally disubstituted at the saturated *meso* position are remarkably stable under ambient conditions, a key consideration for biomedical and technological applications.^15^

**Chart 1:**
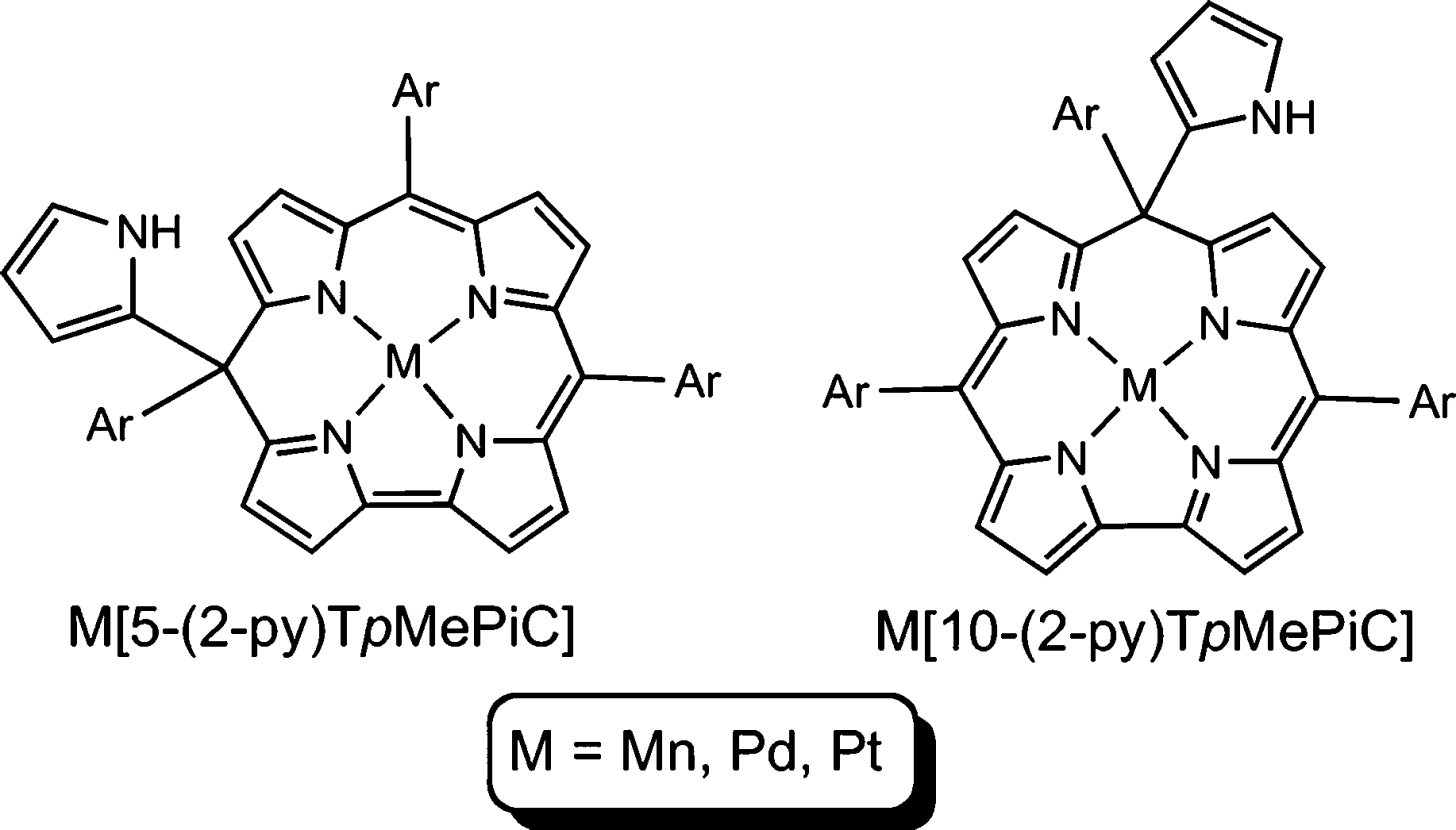
Compounds studied in this work (Ar = 4-methylphenyl).

Recently, we reported exceptionally facile “one-minute” synthesis of pyrrole-appended isocorroles.^16,17^ The present study is aimed at facilitating their applications, especially in the biomedical arena. Key results obtained here include (a) an optimization of the synthesis and isolation of a free-base isocorrole, (b) palladium, platinum, and manganese insertion, (c) photophysical characterization of the resulting complexes, and (d) measurements of the complexes’ ability to sensitize singlet oxygen formation. All the complexes exhibit weak NIR phosphorescence under ambient conditions but sensitize singlet oxygen formation with good quantum yields ranging from 21 to 52% (Chart 1).

## Results and Discussion

### Synthesis and Characterization

Metal insertion into isocorroles has been comparatively less explored relative to that for porphyrins^18,19^ and corroles.^20−24^ Also, certain isocorroles are more delicate and less able to tolerate some of the harsher metal insertion protocols that are applicable to porphyrins and corroles.^7,25^ Using 5/10-(2-pyrrolyl)-5,10,15-tris(*p*-methylphenyl)isocorrole as the model substrate, we describe herein carefully optimized conditions for the insertion of Pd(II), Pt(II), and Mn(II) ions. In each reaction, an unseparated mixture of free-base 5- and 10-(2-pyrrolyl)-5,10,15-tris(*p*-methylphenyl)isocorrole was used as the substrate, followed by separation of the regioisomeric metal complexes, a protocol that we found simpler than one involving separation of the regioisomeric free bases. All the metal complexes yielded clean high-resolution electrospray ionization mass spectra; the diamagnetic Pd and Pt complexes also yielded ^1^H NMR spectra that could be fully assigned.

Perhaps not surprisingly, Pd insertion proved to be the simplest.^15,26^ An olive-green solution of the free-base isocorrole and Pd(II) acetate in DMF was heated to reflux (153 °C), whereupon the color changed immediately to yellowish-brown, accompanied by significant changes in the UV–vis–NIR spectra. Cooling the solution right away to room temperature led to the isolation of the 5-regioisomeric Pd isocorrole in low yields (∼15%). Somewhat milder conditions helped: PdCl_2_ in DMF at 100 °C over 3 h led to the 5- and 10-regioisomeric Pd isocorroles in 49.0 and 2.7% yields, respectively.

Given the exceptional difficulty of inserting kinetically inert platinum ions into corroles,^27,28^ Pt insertion into isocorrole was also expected to be tricky, as it indeed was. Heating a benzonitrile solution of free-base isocorrole and PtCl_2_ to 100 °C for several hours led to no reaction, as judged by mass spectrometric analysis. Increasing the temperature to 180 °C led to chlorinated free-base products, as judged by isotope patterns in the mass spectra, but the products eluted poorly on a column and proved impossible to separate. The use of basic solvents such as refluxing pyridine and mixtures of *o*-dichlorobenzene and isoquinoline at 150 °C also proved fruitless but led to improved survival of the isocorrole starting material, indicating a potentially deleterious effect of HCl on the stability of the isocorrole. Replacing the PtCl_2_ with tetranuclear platinum acetate, [Pt(OAc)_2_]_4_, the reagent of choice for Pt insertion into corroles, and refluxing in benzonitrile for several hours again failed to evince any sign of Pt insertion. Microwave irradiation of the same mixture at 200 °C for 4 h proved similarly uneventful, while attempted reaction at 250 °C led to elimination of the pyrrole sidechain and the isolation of free-base tris(4-methylphenyl)corrole. Platinum insertion was finally accomplished with *cis*-Pt(PhCN)_2_Cl_2_ and sodium acetate in refluxing chlorobenzene, which led to the 5- and 10-regioisomeric Pt(II) isocorrole complexes in 13.3 and 5.1% yields, respectively.

Manganese insertion also proved tricky,^29^ although not quite as severely as platinum insertion. Interaction of the free-base isocorrole with Mn(OAc)_2_·4H_2_O in pyridine at 100 °C (the standard conditions for Mn insertion into porphyrins and corroles) led to low yields (generally under 5%) of the desired Mn(II) product, but also to large amounts of unreacted isocorrole. Prolonged reaction times also led to elimination of the pyrrole sidechain and isolation of an Mn(III) corrole, as judged by UV–vis spectroscopy and mass spectrometry. We surmised—correctly in retrospect—that a different solvent and milder reaction conditions might lead to higher yields of the desired Mn(II) product. Thus, a solution of free-base isocorrole and Mn(OAc)_2_·4H_2_O in CHCl_3_/MeOH, upon refluxing overnight, led to the 5- and 10-regioisomeric Mn(II) products in 38.5 and 2.7% yields, respectively. The regioisomeric nature of the two products, brown and red in color, respectively, was initially unclear since the paramagnetic nature of the compounds precluded ^1^H NMR assignments. However, repeating the synthesis with pre-separated regioisomeric free-base isocorroles identified the brown and red Mn(II) complexes as the 5- and 10-regioisomers, respectively.

**Figure 1:**
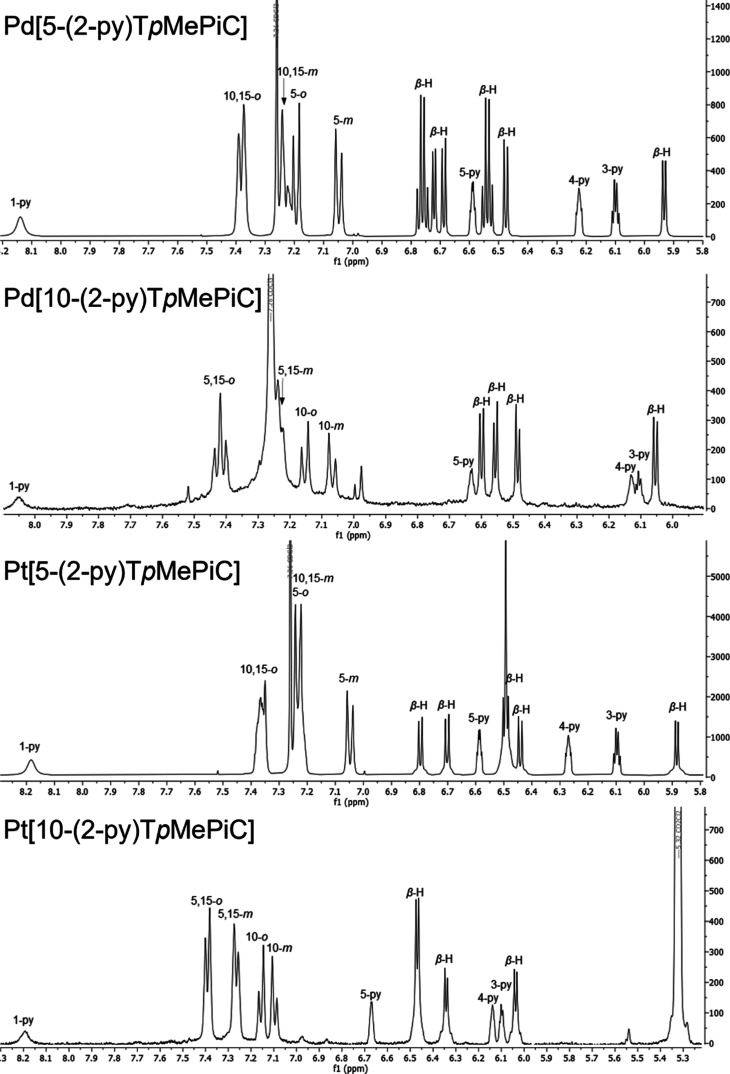
^1^H NMR of new Pd and Pt complexes synthesized.

**Figure 2:**
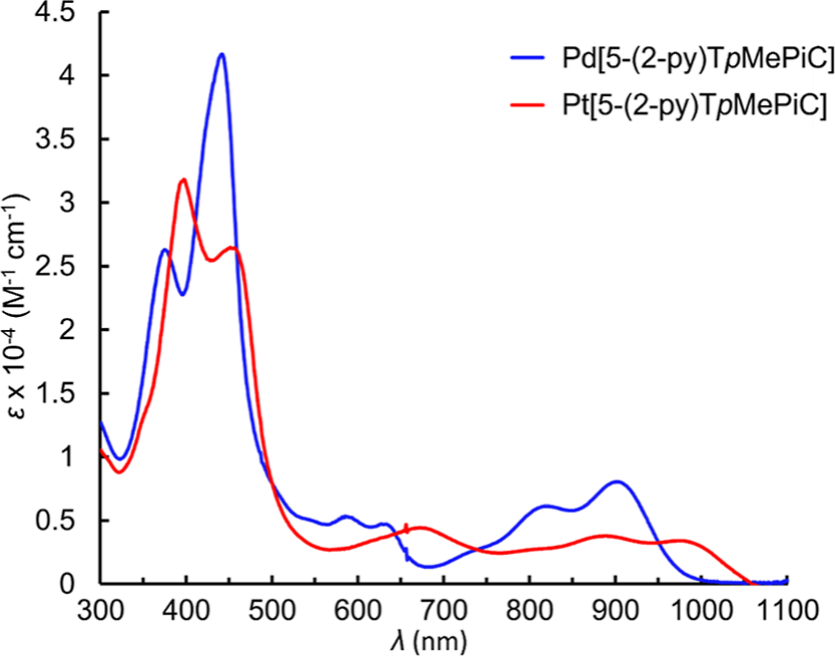
UV–visible spectra of Pd[5-(2-py)T*p*MePiC] and Pt[5-(2-py)T*p*MePiC].

**Figure 3:**
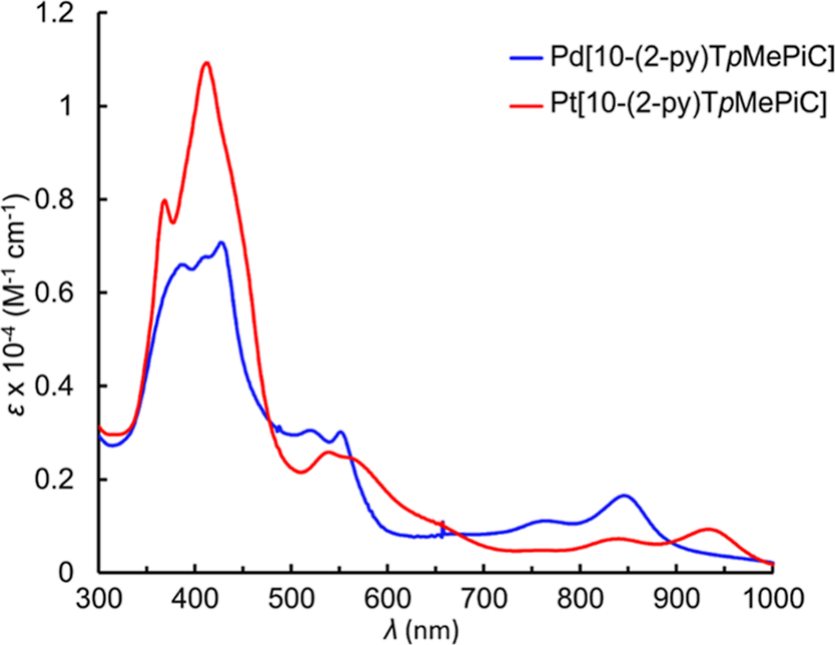
UV–visible spectra of Pd[10-(2-py)T*p*MePiC] and Pt[10-(2-py)T*p*MePiC].

**Figure 4:**
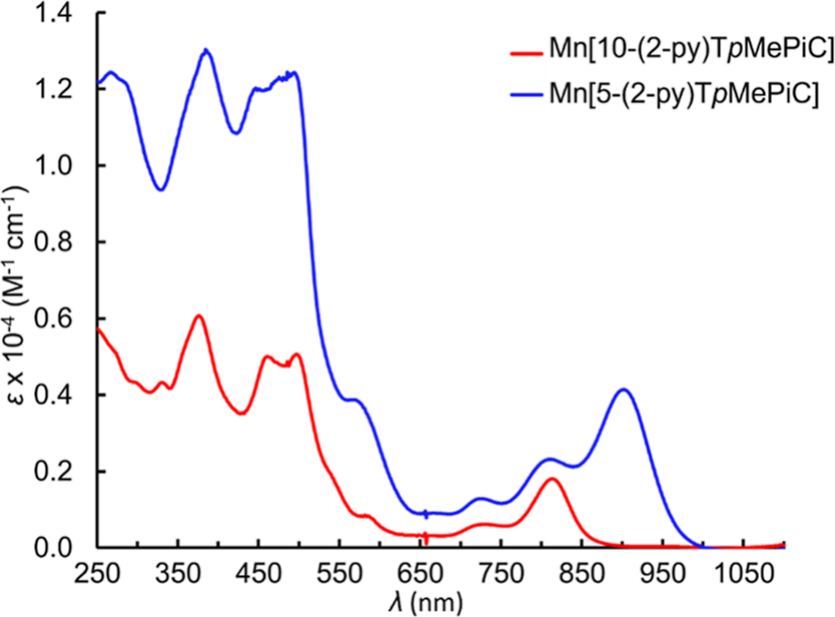
UV–visible spectra of Mn[5-(2-py)T*p*MePiC] and Mn[10-(2-py)T*p*MePiC].

**Figure 5:**
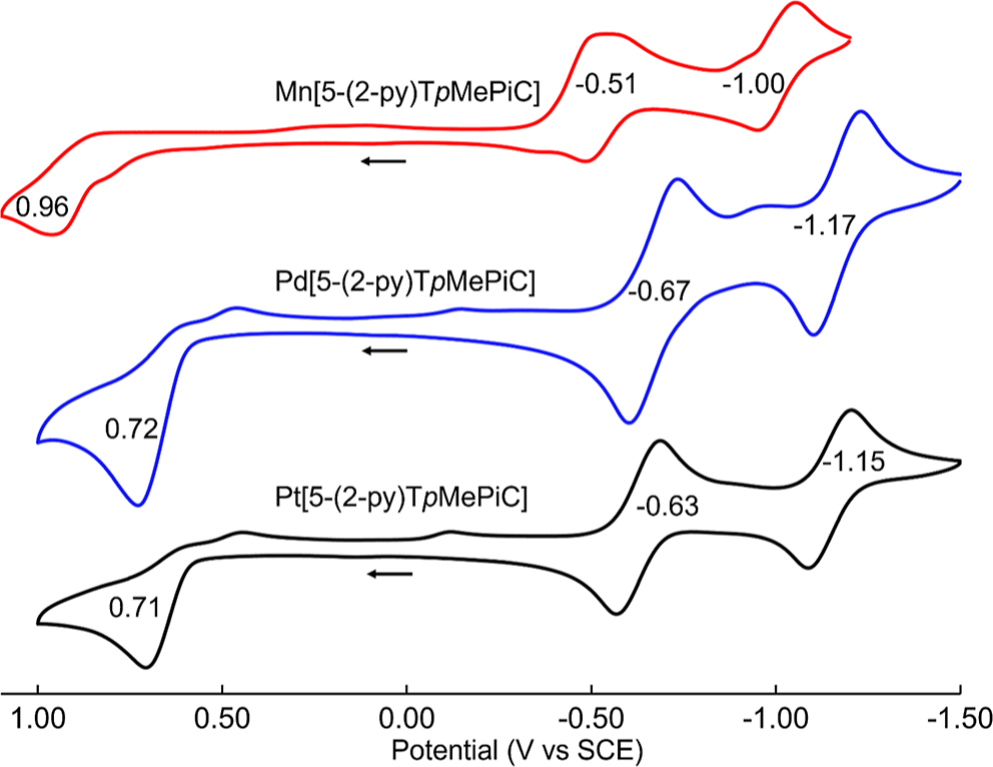
Cyclic voltammograms of M[5-(2-py)T*p*MePiC] (M = Mn, Pd, Pt) in CH_2_Cl_2_ containing 0.1 M TBAP; scan rate 100 mV·s^–1^.

In general, proof of composition and purity was obtained from electrospray ionization mass spectra (see the Supporting Information), ^1^H NMR spectra, and clean thin-layer chromatograms. Note that the 5- and 10-regioisomeric complexes can be readily told apart from the ^1^H NMR spectra, given that only the latter correspond to time-averaged *C*_*s*_ symmetry (Figure 1). All the isocorrole complexes exhibit strong NIR absorption (Figures 2–4), with the 5-isocorrole derivatives absorbing farther into the NIR relative to the 10-isomers (for a given metal ion). The main NIR-absorbing peaks also vary significantly as a function of the coordinated metal (for a given isocorrole isomer). The more abundant 5-regioisomers were also characterized with cyclic voltammetry (Figure 5). Each compound was found to exhibit two reversible reductions and an irreversible oxidation, the latter possibly reflecting oxidation and subsequent deprotonation of the dangling pyrrole substituents. Even in the absence of reversible oxidations, the relatively high reduction potentials indicate a low-energy LUMO, relative to closed-shell metalloporphyrins and metallocorroles,^21,23,30,31^ and hence smaller HOMO–LUMO gaps, consistent with NIR absorption by the compounds.

### Photophysical Studies

Although there are extensive studies of visible and NIR absorption in the literature, little has been reported on the emission properties of the isocorroles. The only reported NIR emission of a related species involves 10-silacorroles^32^ and an extended isocorrole,^33^ and both exhibit emission within the 700–1200 nm range. The compounds reported here all exhibit broad single-peak NIR phosphorescence between 850 and 1500 nm when excited with a 405 nm LED excitation light source (Table 1).

**Table 1:** Summary of Emission, Lifetime, and Quantum Yield in Anoxic Dichloromethane^a^

compounds	NIR emission maxima (nm)	lifetime (μs)	phosphorescence quantum yield (%)	singlet oxygen quantum yield (%)
Pt[5-(2-py)T*p*MePiC]	1072	43	—	24
Pt[10-(2-py)T*p*MePiC]	962	27	—	31
Pd[5-(2-py)T*p*MePiC]	1000	26	0.10	34
Pd[10-(2-py)T*p*MePiC]	958	27	0.04	52
Mn[5-(2-py)T*p*MePiC]	965	23	—	21
Mn[10-(2-py)T*p*MePiC]	959	28	—	33

a “—”: too weak to be determined by the relative method.

**Figure 6:**
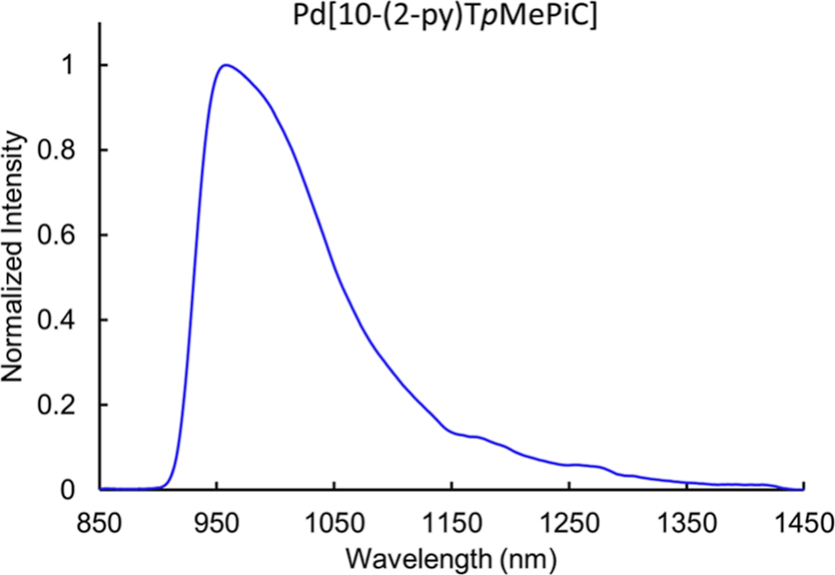
Phosphorescence spectrum (λ_ex_ = 405 nm) of Pd[10-(2-py)T*p*MePiC] at 1.0 × 10^–4^ M in anoxic dichloromethane. 1 s integration time, bandpass 26 nm, average of 5 scans.

In the 5-pyrrolyl series, a significant shift in the emission maxima was observed (959, 1000, and 1072 nm for Mn, Pd, and Pt, respectively), implicating metal-based orbitals in the luminescent transition. As expected, the lifetimes increase from 23 to 26, and to 43 μs going from Mn to Pd, and to Pt, respectively, consistent with increased spin–orbit coupling due to the heavy atom effect. Interestingly, the phosphorescence quantum yield, which reflects a balance of multiple photophysical pathways, was found to be the highest for Pd, at 0.10%, whereas it was too weak to be reliably determined for Mn and Pt (Figure 6).

In the 10-pyrrolyl series, no significant differences in the emission maxima were observed among the three metals (959, 958, and 962 nm for Mn, Pd, and Pt, respectively), which are similar to the emission maximum of the free ligand. In addition, the luminescence lifetimes were found to be identical within experimental error (27 μs). This observation suggests that the luminescence is largely ligand-centered, with minimum involvement of metal-based orbitals. Some metal involvement can still be inferred though because the luminescence intensity follows the same trend as the 5-py series, with the Pd complex being the most emissive and some broadening being observed in the emission profile (see Supporting Information).

### Singlet Oxygen Sensitization

**Figure 7:**
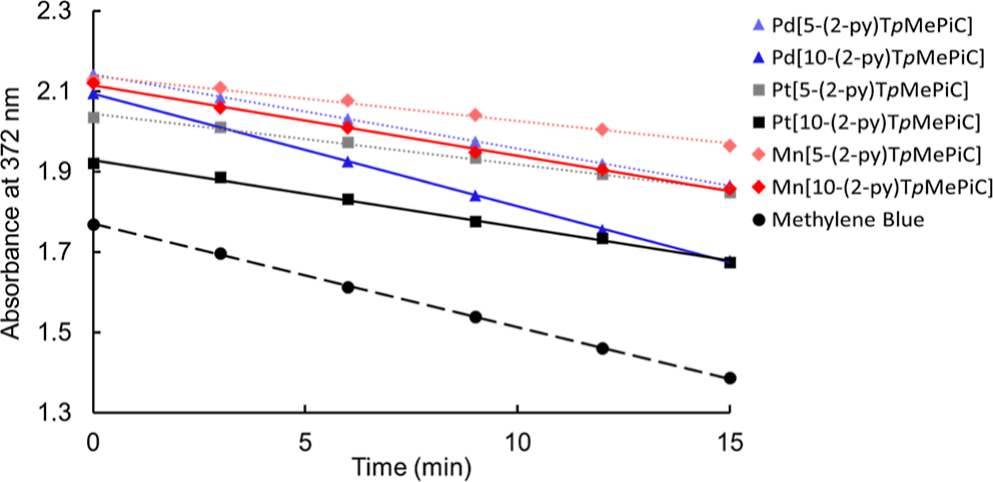
Degradation profile of 9,10-diphenylanthracene (0.28 mM) in an air-saturated solution (9:1 EtOH/THF) in the presence of the isocorroles (5 μM) upon irradiation with a 405 nm LED.

The ability of a compound to efficiently sensitize singlet oxygen formation makes it useful for applications in photodynamic therapy of cancer and certain other diseases.^34−38^ Although the metalloisocorroles examined show low NIR phosphorescence quantum yields (compared, for example, to metalloporphyrins^39−41^ and metallocorroles^42−44^), they were found to efficiently sensitize singlet oxygen formation. Singlet oxygen quantum yields were determined by a chemical method using 9,10-diphenylanthracene as the singlet oxygen acceptor and methylene blue as the reference dye (φ = 48%, Figure 7).^45,46^ All the complexes were found to sensitize singlet oxygen formation with singlet oxygen quantum yields in the range 21–52%, as shown in Table 1. The efficiency of metalloisocorroles as singlet oxygen sensitizers was found to be comparable to that of free-base corroles (34–86%).^47^ They are more efficient than Ir(III) corroles as singlet oxygen sensitizers^48,49^ but less so than Ga(III) (51–77%)^50^ and Os^IV^N corroles(76–95%).^46^

For each metal, the 10-py complex was found to be a stronger singlet oxygen sensitizer than the 5-py complex. For the Pd complexes, Pd[10-(2-py)T*p*MePiC] was found to exhibit a singlet oxygen quantum yield of 52%, the highest among the metalloisocorroles studied, compared to 34% for Pd[5-(2-py)T*p*MePiC]. Likewise, for the Pt complexes, Pt[10-(2-py)T*p*MePiC] exhibits a singlet oxygen quantum yield of 31% compared to 24% for Pt[5-(2-py)T*p*MePiC]. Along with their NIR absorption, their strong singlet oxygen-sensitizing ability, bodes well for potential applications of metalloisocorroles for photodynamic therapy.

## Conclusions

Isocorroles have attracted attention in recent years as comparatively stable, NIR-absorbing tautomers of corroles. Their emission properties, however, have remained unexplored until now. Herein, we described methods for the insertion of Mn(II), Pd(II), and Pt(II) into the isocorrole ring system. The products are weakly phosphorescent in the NIR under ambient conditions, with a maximum observed phosphorescence quantum yield of 0.1% observed for one of the Pd complexes. All the compounds, however, are good singlet oxygen sensitizers, with singlet oxygen quantum yields of 21–52%. The finding, along with compounds’ NIR-absorbing properties, suggests potential applications as photosensitizers for photodynamic therapy.

## Experimental Section

### Materials

All reagents were purchased from Sigma-Aldrich and used as received, except for pyrrole, which was distilled and stored in the freezer. Silica gel 60 (0.04–0.063 mm particle size, 230–400 mesh, Merck) was employed for flash chromatography. Silica gel 60 preparative thin-layer chromatographic plates (20 cm × 20 cm × 0.5 mm, Merck) were used for final purification of all compounds.

### General Instrumental Methods

UV–visible spectra were recorded on an HP 8454 spectrophotometer. ^1^H NMR spectra were recorded on a 400 MHz Bruker AVANCE III HD spectrometer equipped with a 5 mm BB/1H SmartProbe and referenced to either residual CH_2_Cl_2_ at 5.32 ppm or residual CHCl_3_ at 7.26 ppm. High-resolution electrospray-ionization mass spectra were recorded on an Orbitrap Exploris 120 spectrometer using methanolic solutions.

Cyclic voltammetry was carried out at ambient temperature with a Gamry Reference 620 potentiostat equipped with a three-electrode system: a glassy carbon working electrode, a platinum wire counter electrode, and a saturated calomel reference electrode (SCE). Tetra(*n*-butyl)ammonium perchlorate (*CAUTION!*) was used as the supporting electrolyte. Anhydrous CH_2_Cl_2_ (Aldrich) was used as the solvent. The electrolyte solution was purged with argon for at least 2 min prior to all measurements, which was carried out under an argon blanket. All potentials were referenced to the SCE.

### Synthesis of H_2_[5/10-(2-py)T*p*MePiC]

To a stirred solution of 5,10,15-tris(*p*-methylphenyl)corrole, H_3_[T*p*MePC]^51,52^ (132.9 mg, 0.23 mmol), in dichloromethane (25 mL), were added DDQ (79.4 mg, ∼1.5 equiv) and pyrrole (0.16 mL, ∼10 equiv) in succession, whereupon the solution, within seconds, turned olive-green. The solvent was carefully evaporated under vacuum at ambient temperature and the residue purified by silica column using dichloromethane/*n*-hexane 1:1. The first green fraction was collected (NOTE! The colorless eluate that preceded the green fraction contained excess pyrrole and was discarded) and the solvents evaporated to give pure free-base isocorrole as a mixture of isomers. Yield (mixture of 5- and 10-regiosiomers): 119 mg (80.4%). When needed, the regioisomeric free-base isocorroles were separated with preparative thin-layer chromatography using acetone/*n*-hexane 1:8 as the mobile phase (which is a significant improvement over the conditions reported previously^16^).

### Synthesis of Pd[5/10-(2-py)T*p*MePiC]

A solution of free-base isocorrole (34.7 mg, 0.055 mmol) and PdCl_2_ (19 mg, ∼2 equiv) in 10 mL of DMF was heated to 100 °C under argon. After 3 h, the reaction mixture was cooled down, the solvent was evaporated, and the residue purified by silica column using dichloromethane/*n*-hexane 1:3. The first red and yellow fractions were collected (the two fractions were collected as one because thin-layer chromatography showed considerable overlap). The solvents were evaporated and the residue purified by preparative thin-layer chromatography using dichloromethane/*n*-hexane 1:1. The red and yellow fractions separated on the plate and were each purified further individually by preparative thin-layer chromatography using dichloromethane/*n*-hexane 1:6 to give 19.8 mg (49%) and 1.1 mg (2.7%) of the Pd-complexes of the 5-isomer (yellow fraction) and the 10-isomer (red fraction), respectively. Analytical details are as follows.

#### Pd[5-(2-py)T*p*MePiC]

UV–vis (CH_2_Cl_2_): λ_max_ (nm), [ε x 10^–4^ (M^–1^ cm^–1^)]: 375 (2.63), 441 (4.17), 585 (0.53), 627 (0.47), 820 (0.61), 901 (0.80). ^1^H NMR (400 MHz, CDCl_3_, δ): 8.14 (s, 1H, 1-pyrrolyl), 7.42–7.34 (m, 4H, 10,15-*o*-Ph), 7.26–7.22 (m, 4H, 10,15-*m*-Ph), 7.19 (d, *J* = 8.1 Hz, 2H, 5-*o*-Ph), 7.05 (d, *J* = 8.1 Hz, 2H, 5-*m*-Ph), 6.78–6.73 (m, 2H, β-H), 6.72 (d, *J* = 3.8 Hz, 1H, β-H), 6.69 (d, *J* = 4.6 Hz, 1H, β-H), 6.59 (td, *J* = 2.7, 1.5 Hz, 1H, 5-pyrrolyl), 6.57–6.50 (m, 2H, β-H), 6.48 (d, *J* = 4.6 Hz, 1H, β-H), 6.26–6.19 (m, 1H, 4-pyrrolyl), 6.10 (q, *J* = 2.9 Hz, 1H, 3-pyrrolyl), 5.93 (d, *J* = 3.8 Hz, 1H, β-H), 2.44 (s, 6H, 10,15-*p*-CH_3_), 2.32 (s, 3H, 5-*p*-CH_3_). MS (ESI, positive mode): *m*/*z* calcd for C_44_H_33_N_5_PdH, 738.1856; [M + H^+^] found, 738.1859.

#### Pd[10-(2-py)T*p*MePiC]

UV–vis (CH_2_Cl_2_): λ_max_ (nm), [ε × 10^–4^ (M^–1^ cm^–1^)]: 385 (0.66), 426 (0.71), 518 (0.31), 551 (0.30), 765 (0.11), 845 (0.16). ^1^H NMR (400 MHz, CDCl_3_, δ): 8.05 (s, 1H, 1-pyrrolyl), 7.46–7.37 (m, 4H, 5,15-*o*-Ph), 7.26–7.20 (m, 4H, 5,15-*m*-Ph), 7.15 (d, *J* = 8.1 Hz, 2H, 10-*o*-Ph), 7.07 (d, *J* = 8.1 Hz, 2H, 10-*m*-Ph), 6.64–6.62 (m, 1H, 5-pyrrolyl), 6.60 (d, *J* = 4.5 Hz, 2H, β-H), 6.56 (d, *J* = 4.2 Hz, 2H, β-H), 6.49 (d, *J* = 4.3 Hz, 2H, β-H), 6.14–6.09 (m, 2H, overlapping 4-pyrrolyl and 5-pyrrolyl), 6.05 (d, *J* = 4.4 Hz, 2H, β-H), 2.44 (s, 6H, 5,15-*p*-CH_3_), 2.32 (s, 3H, 10-*p*-CH_3_). MS (ESI, positive mode): *m*/*z* calcd for C_44_H_33_N_5_PdH, 738.1855; [M + H^+^] found, 738.1859.

### Synthesis of Pt[5/10-(2-py)T*p*MePiC]

A solution of free-base isocorrole (25.4 mg, 0.04 mmol), Pt(PhCN)_2_Cl_2_ (28.8 mg, ∼1.5 equiv), and NaOAc (14.6 mg, ∼4 equiv) in 10 mL of chlorobenzene was heated to reflux under argon. After 3 h, the reaction mixture was cooled down, the solvent was evaporated, and the residue was purified by a silica column using dichloromethane/*n*-hexane 1:1. All fractions were collected, the solvents were evaporated, and the residue was purified by preparative thin-layer chromatography using ethyl acetate/*n*-hexane 1:10. An overlapping red and yellow band with Rf = 0.51–0.63 was isolated, corresponding to the Pt-complexes as a mixture of isomers. To separate the isomers, preparative thin-layer chromatography using dichloromethane/*n*-pentane 1:3 was used and the Pt-complexes of the 5-isomer (yellow band) and 10-isomer (red band) were isolated in yields of 4 mg (11.9%) and 1.7 mg (5.1%), respectively. Analytical details are as follows.

#### Pt[5-(2-py)T*p*MePiC]

UV–vis (CH_2_Cl_2_): λ_max_ (nm), [ε × 10^–4^ (M^–1^ cm^–1^)]: 397 (3.18), 451 (2.65), 672 (0.44), 887 (0.38), 977 (0.34). ^1^H NMR (400 MHz, CDCl_3_, δ): 8.11 (s, 1H, 1-pyrrolyl), 7.34–7.26 (m, 4H, 10,15-*o*-Ph), 7.18–7.12 (m, 6H, overlapping 10,15-*m*-Ph and 5-*o*-Ph), 6.98 (d, *J* = 8.0 Hz, 2H, 5-*m*-Ph), 6.73 (d, *J* = 4.8 Hz, 1H, β-H), 6.63 (d, *J* = 4.9 Hz, 1H, β-H), 6.52 (td, *J* = 2.7, 1.5 Hz, 1H, 5-pyrrolyl), 6.44–6.40 (m, 4H, β-H), 6.37 (d, *J* = 4.9 Hz, 1H, β-H), 6.20 (ddd, *J* = 4.0, 2.7, 1.5 Hz, 1H, 4-pyrrolyl), 6.03 (q, *J* = 2.9 Hz, 1H, 3-pyrrolyl), 5.81 (d, *J* = 3.9 Hz, 1H, β-H), 2.36 (s, 6H, 10,15-*p*-CH_3_), 2.25 (s, 3H, 5-*p*-CH_3_). MS (ESI, positive mode): *m*/*z* calcd for C_44_H_33_N_5_PtH, 827.2460; [M + H^+^] found, 827.2461.

#### Pt[10-(2-py)T*p*MePiC]

UV–vis (CH_2_Cl_2_): λ_max_ (nm), [ε × 10^–4^ (M^–1^ cm^–1^)]: 368 (0.80), 411 (1.10), 539 (0.26), 839 (0.07), 933 (0.09). ^1^H NMR (400 MHz, CD_2_Cl_2_, δ): 8.19 (s, 1H, 1-pyrrolyl), 7.39 (d, *J* = 7.5 Hz, 4H, 5,15-*o*-Ph), 7.27 (d, *J* = 7.5 Hz, 4H, 5,15-*m*-Ph), 7.16 (d, *J* = 8.3 Hz, 2H, 10-*o*-Ph), 7.10 (d, *J* = 8.2 Hz, 2H, 10-*m*-Ph), 6.69–6.65 (m, 1H, 5-pyrrolyl), 6.47 (d, *J* = 4.6 Hz, 4H, β-H), 6.34 (d, *J* = 4.4 Hz, 2H, β-H), 6.15–6.12 (m, 1H, 4-pyrrolyl), 6.12–6.08 (m, 1H, 3-pyrrolyl), 6.04 (d, *J* = 4.7 Hz, 2H, β-H), 2.44 (s, 6H, 5,15-*p*-CH_3_), 2.32 (s, 3H, 10-*p*-CH_3_). MS (ESI, positive mode): *m*/*z* calcd for C_44_H_33_N_5_PtH, 827.2460; [M + H^+^] found, 827.2464.

### Synthesis of Mn[5/10-(2-py)T*p*MePiC]

A solution of free-base isocorrole (17 mg, 0.027 mmol) in CHCl_3_ (5 mL) and MeOH (5 mL) was heated to reflux, and to it was added Mn(OAc)_2_·4H_2_O (30.5 mg, ∼5 equiv) in MeOH (1 mL). The resulting mixture was refluxed overnight, cooled down, the solvents were evaporated, and the residue was purified by silica column chromatography. Dichloromethane eluated impurities, after which the polarity was increased to ethyl acetate/MeOH 2:1 to eluate a brown fraction that corresponded to the Mn-complex as a mixture of isomers. The isomers were separated by preparative thin-layer chromatography using ethyl acetate/*n*-hexane 1:2 with 1% acetic acid as the solvent. A red band moved in front of a brown band, corresponding to the 10- and 5-isomers of the Mn-complex, respectively. If the isomers did not fully separate, the top of the band containing the red fraction and some of the brown fraction was isolated and purified again by preparative thin-layer chromatography using the same solvents. The process was repeated until the two bands fully separated. Yields and analytical details are as follows.

#### Mn[5-(2-py)T*p*MePiC]

The yield of the product was 7.1 mg (38.5%). UV–vis (CH_2_Cl_2_): λ_max_ (nm), [ε × 10^–4^ (M^–1^ cm^–1^)]: 266 (1.25), 384 (1.30), 485 (1.24), 725 (0.13), 810 (0.23), 901 (0.41). MS (ESI, positive mode): *m*/*z* calcd for C_44_H_33_N_5_MnH, 687.2189; [M + H^+^] found, 687.2174.

#### Mn[10-(2-py)T*p*MePiC]

The yield of the product was 0.5 mg (2.7%). UV–vis (CH_2_Cl_2_): λ_max_ (nm), [ε × 10^–4^ (M^–1^ cm^–1^)]: 330 (0.43), 376 (0.61), 461 (0.50), 496 (0.51), 581 (0.09), 730 (0.06), 813 (0.18). MS (ESI, positive mode): *m*/*z* calcd for C_44_H_33_N_5_Mn, 686.2111; [M^+^] found, 686.2112.

### Photophysical Measurements

All photophysical studies were performed in sealed cuvettes under dry N_2_ using degassed dichloromethane. Absorption spectra (for quantum yield) were recorded on a HORIBA Duetta spectrophotometer using HORIBA EzSpec software. Phosphorescence emissions were measured on an OLIS NIRCPL Solo at 10^–4^ M dichloromethane solutions using a 405 nm LED light source. Visible-region emissions for the free base isocorroles (excitation at 455 nm) were recorded on a HORIBA Duetta spectrophotometer using HORIBA EzSpec software. Quantum yields were determined by a relative method on an OLIS NIR CPL Solo instrument. The instrument is built with a quarter wave plate from Thorlabs; the detector uses InGaAs PIN photodiodes (Hamamatsu) cooled at −25 °C using a thermoelectric cooler. The sample is illuminated with LEDs (Everlight) driven to an output of 1000 mW.

For quantum yield, prior to each measurement, the dye solution in a screw-capped cuvette was unscrewed, diluted to the new concentration, and then purged with nitrogen for 10 min. Yb(tta)_3_(H_2_O)_2_ was used as the reference compound (QY = 0.35% in toluene). Phosphorescence lifetimes were measured in degassed dichloromethane from solutions of 10^–4^ to 10^–6^ M using an OLIS CPL Solo spectrofluorometer; spectra were collected using pulsed excitation at 405 nm and time-resolved emission measurements fixed at the peak of the strongest emission. A first-order exponential decay curve was fit to the collected data to estimate the fluorescence lifetime (τ_obs_). Values are reported as measured lifetimes (observation wavelength).

### Singlet Oxygen Sensitization Measurements

Singlet oxygen quantum yields were calculated by a relative method using 9,10-diphenylanthracene as the singlet oxygen acceptor. The sensitizers (isocorroles) were dissolved to EtOH/THF (9:1 v/v) (5 μM adjusted for identical absorption at the excitation wavelength). Then, a 0.28 mM solution of 9,10-diphenylanthracene in 9:1 v/v EtOH/THF was prepared. Equal volumes of the 9,10-diphenylanthracene solution and each photosensitizer solution were mixed in a 1 cm path length cuvette. The mixture was saturated with oxygen by bubbling air through it for 3 min. The cuvette was sealed, and its absorbance was initially measured before it was irradiated with light (two 405 nm Everlight LEDs each driven to an output of 1000 mW, 10 nm slit) for 3 min. The cuvette was shaken and then, the absorbance spectra were remeasured. This experiment was repeated 4 more times by taking the absorbance measurement at 3 min light irradiation intervals. Singlet oxygen quantum yields were calculated from the slope of the curve (absorbance at 372 nm vs time) using methylene blue as the reference (φ = 0.48).

## Supporting Information Available

ESI mass spectra and selected photophysical data (PDF)

## Notes

All data generated or analyzed in this study are included in this published article and its Supporting Information.
