# Supplementary material for: Transition-Metal Isocorroles as Singlet Oxygen Sensitizers
Source: Inorg Chem. 2023 May 4;62(19):7483–90. doi: 10.1021/acs.inorgchem.3c00782 (PMC10189730; doi:10.1021/acs.inorgchem.3c00782)
Supplement: Supplementary file 1 — ic3c00782_si_001.pdf [file ic3c00782_si_001.pdf]

Supporting Information

Transition Metal Isocorroles as Singlet Oxygen Sensitizers

Simon Larsen,[a] Joseph Adewuyi,[b] Gaël Ung,*[,b] and Abhik Ghosh*[,a]

[a]Department of Chemistry, University of Tromsø, N-9037 Tromsø, Norway

[b]Department of Chemistry, University of Connecticut, 55 N. Eagleville Rd, Storrs, CT 06269, USA

Contents **Page**

Contents	Page
A. ESI-MS spectra	S2
B. Luminescence spectra	S5
C. Phosphorescence lifetime spectra	S10
D. Singlet oxygen sensitization measurements	S13

A. ESI-MS spectra (positive mode)

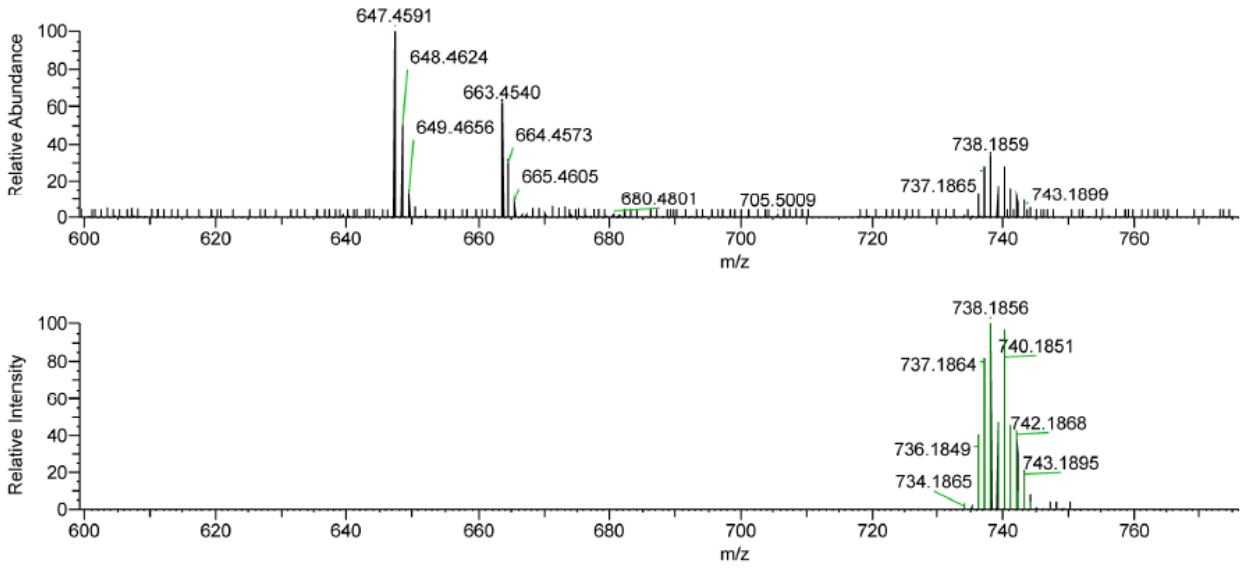

Figure S1. ESI-MS spectrum experimental (top) and simulated (bottom) of Pd[5-(2-py)T*p*MePiC].

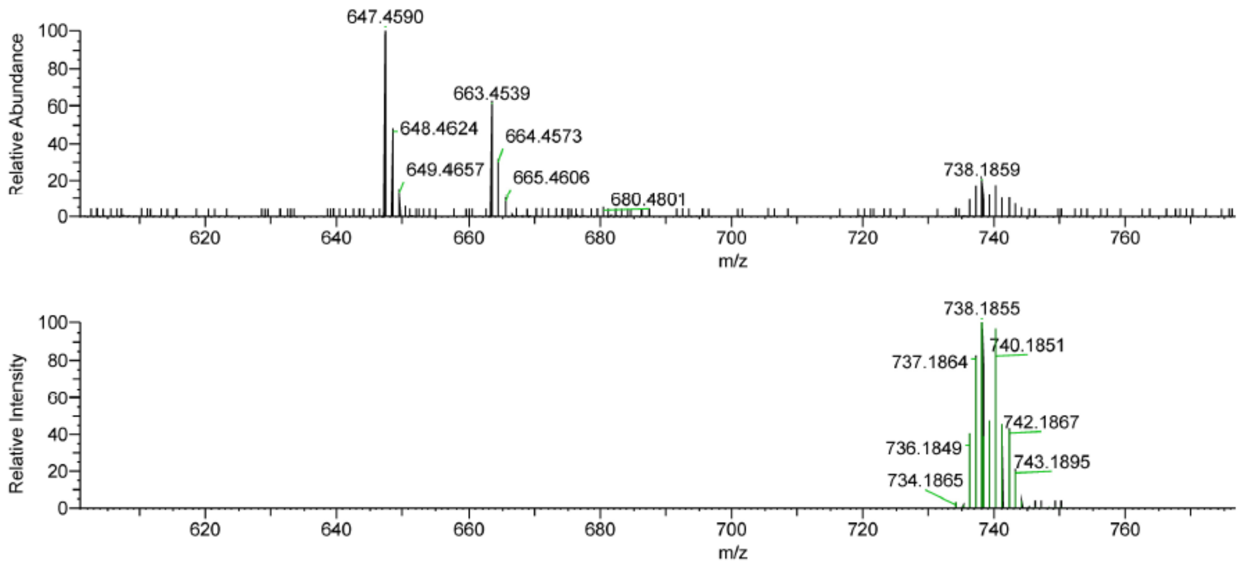

Figure S2. ESI-MS spectrum experimental (top) and simulated (bottom) of Pd[10-(2-py)T*p*MePiC].

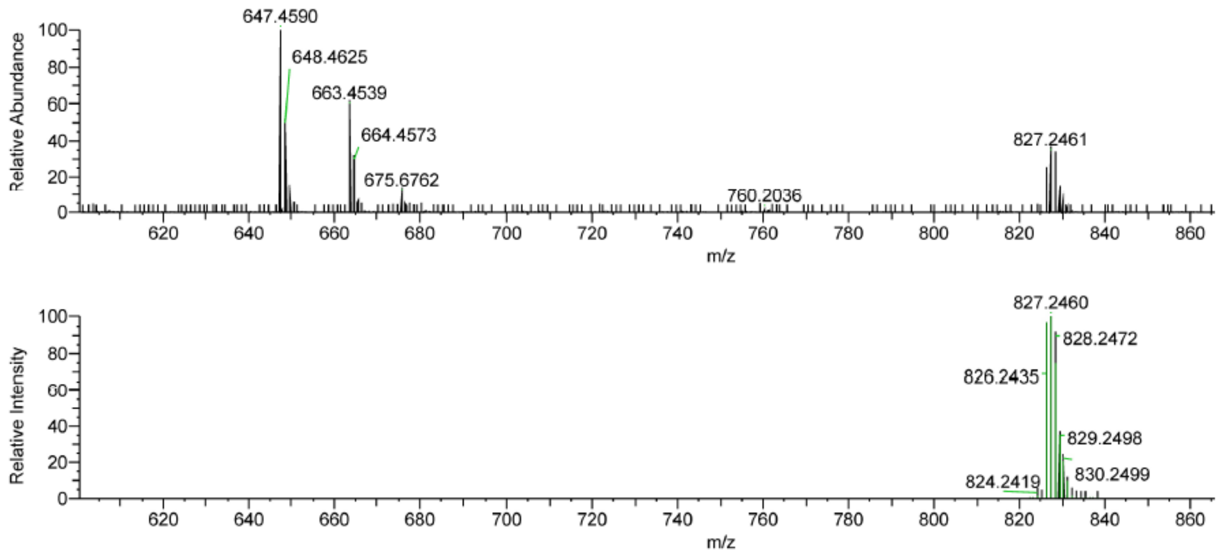

Figure S3. ESI-MS spectrum experimental (top) and simulated (bottom) of Pt[5-(2-py)T*p*MePiC].

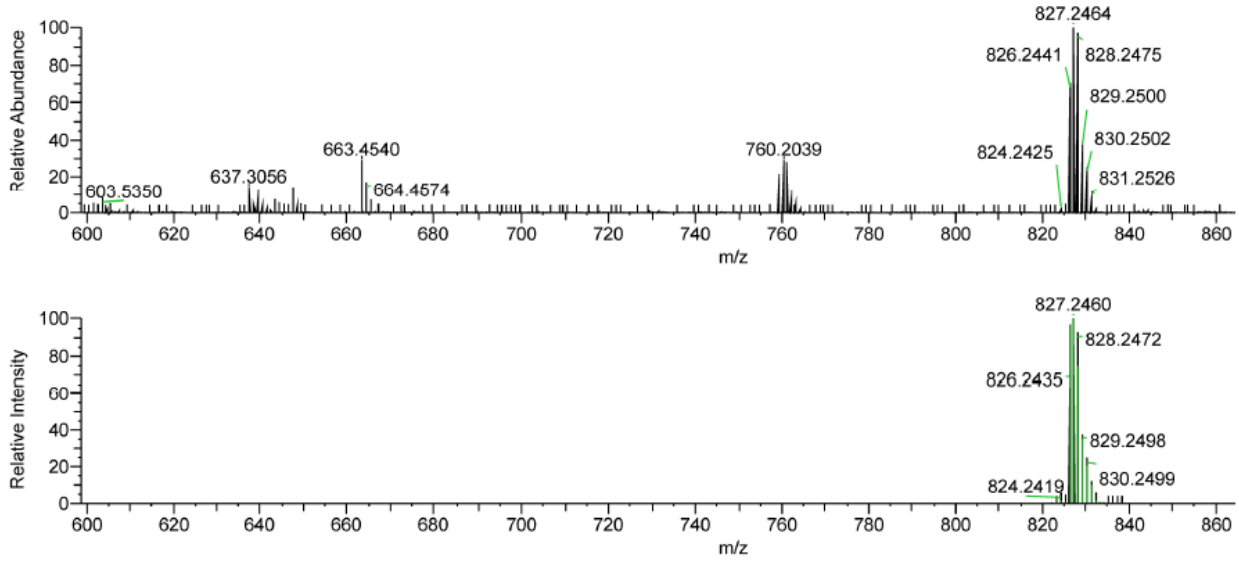

Figure S4. ESI-MS spectrum experimental (top) and simulated (bottom) of Pt[10-(2-py)T*p*MePiC].

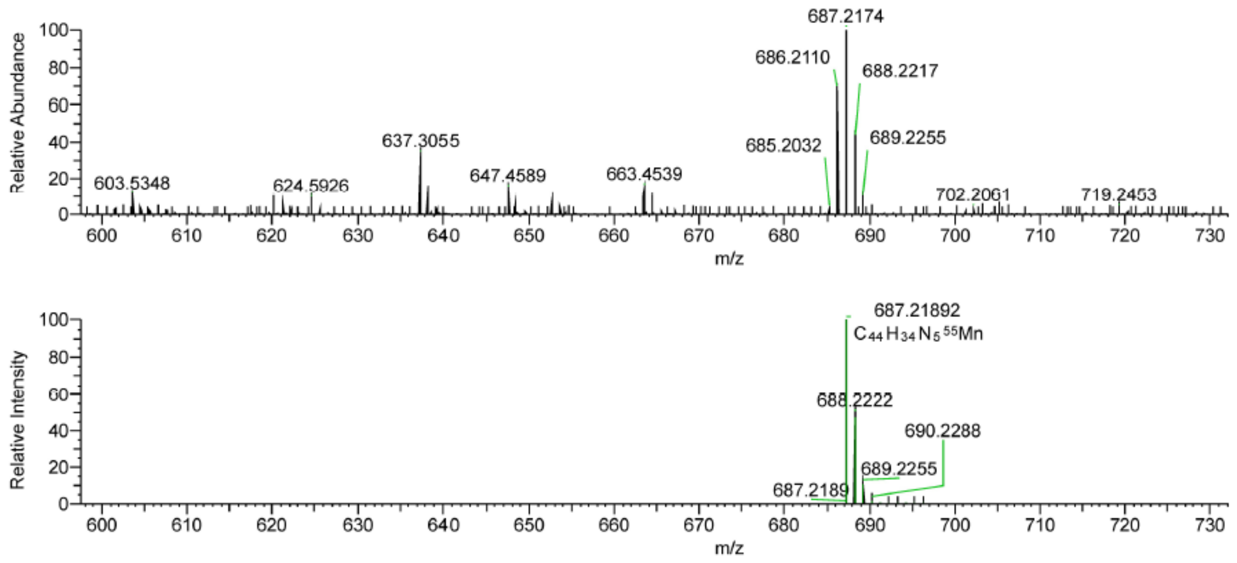

Figure S5. ESI-MS spectrum experimental (top) and simulated (bottom) of Mn[5-(2-py)T*p*MePiC].

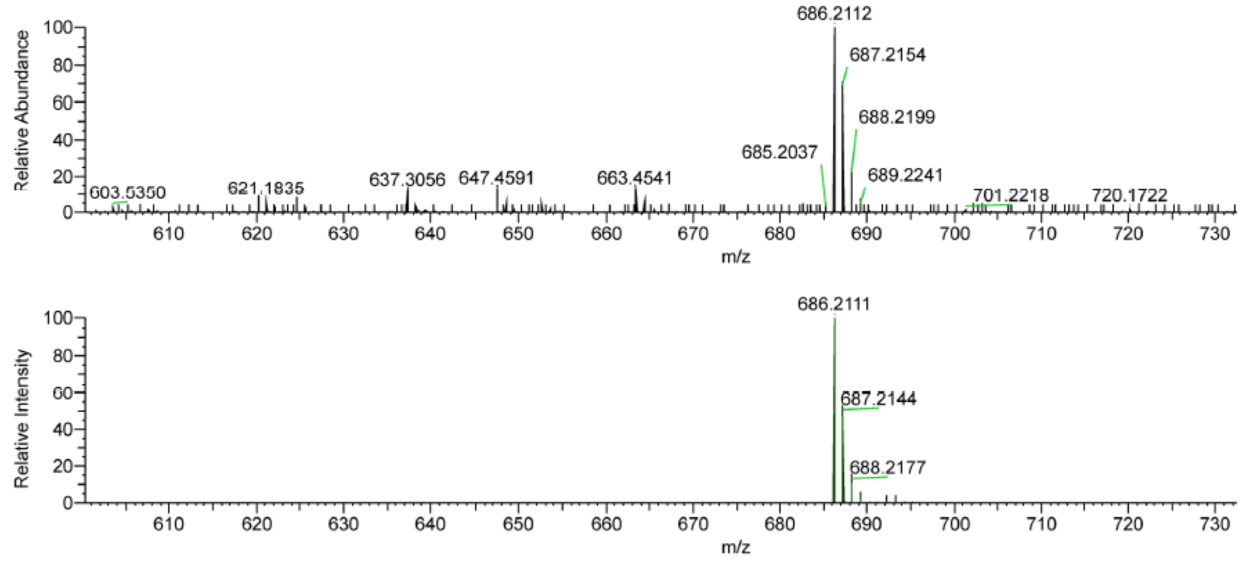

Figure S6. ESI-MS spectrum experimental (top) and simulated (bottom) of Mn[10-(2-py)T*p*MePiC].

B. Luminescence spectra

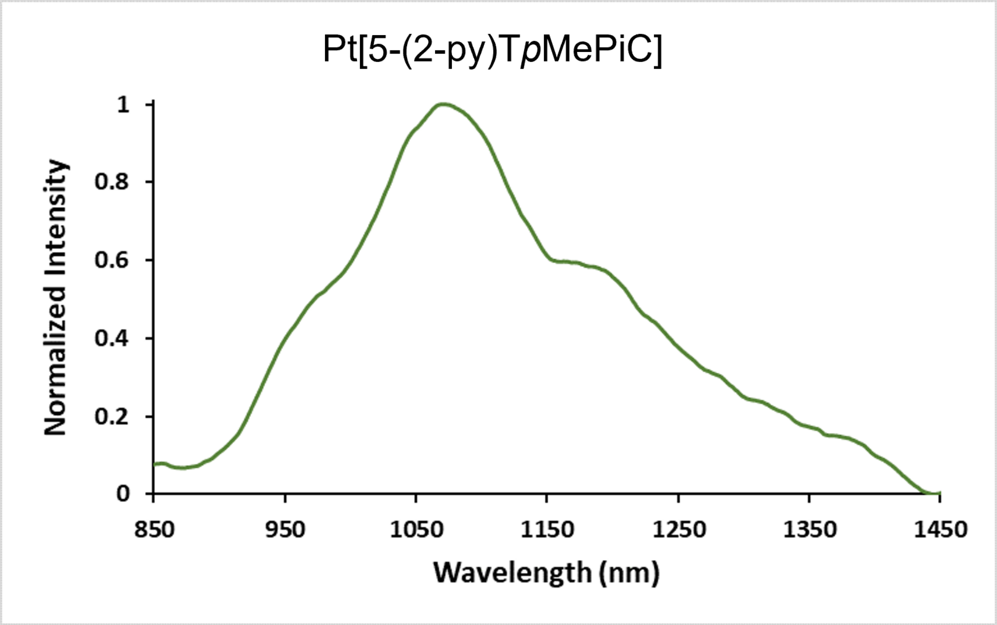

Figure S7: Phosphorescence of Pt[5-(2-py)T*p*MePiC] at 4.0 x 10^{-4} M in anoxic dichloromethane: 1 sec integration time, bandpass 50 nm, average of 5 scans.

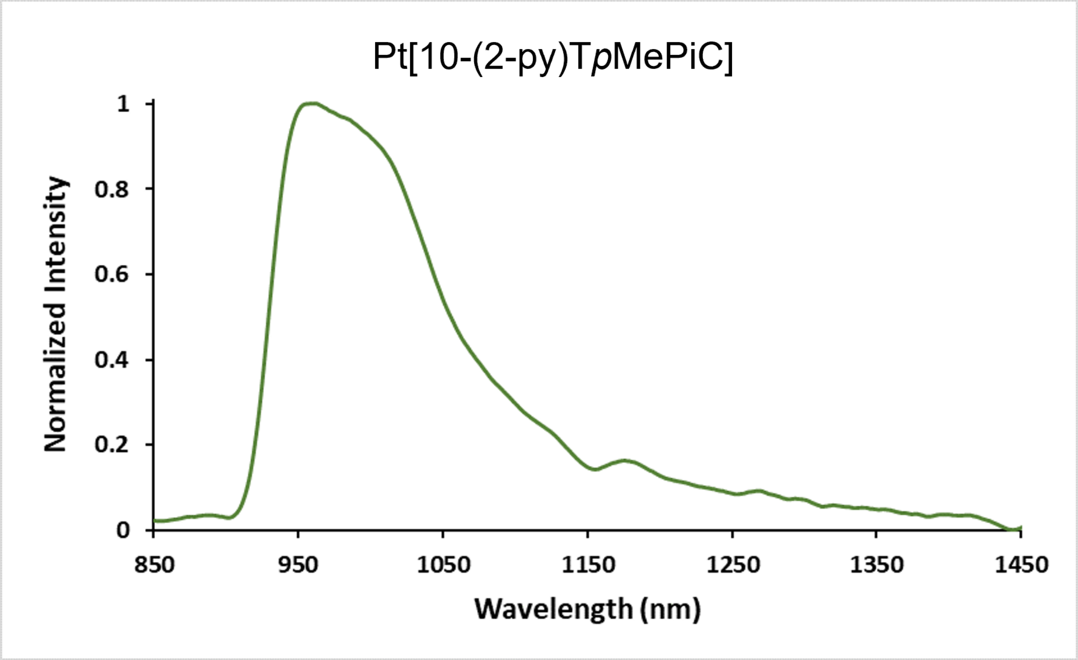

Figure S8: Phosphorescence of Pt[10-(2-py)T*p*MePiC] at 1.2 x 10^{-4} M in anoxic dichloromethane: 1 sec integration time, bandpass 26 nm, average of 5 scans.

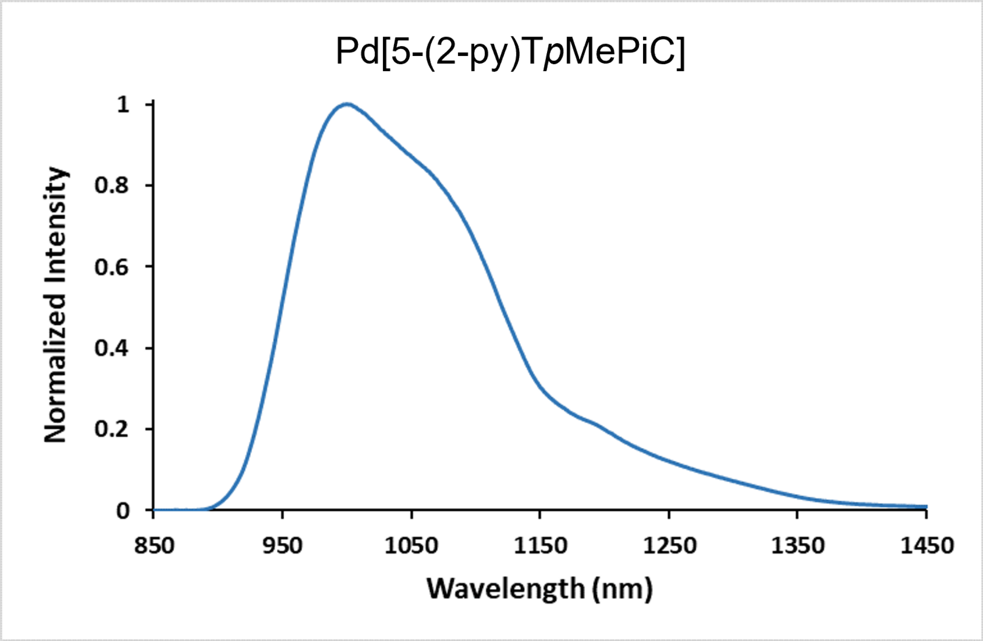

Figure S9: Phosphorescence of Pd[5-(2-py)T*p*MePiC] at 4.0 x 10^{-4} M in anoxic dichloromethane: 0.1 sec integration time, bandpass 50 nm, average of 3 scans.

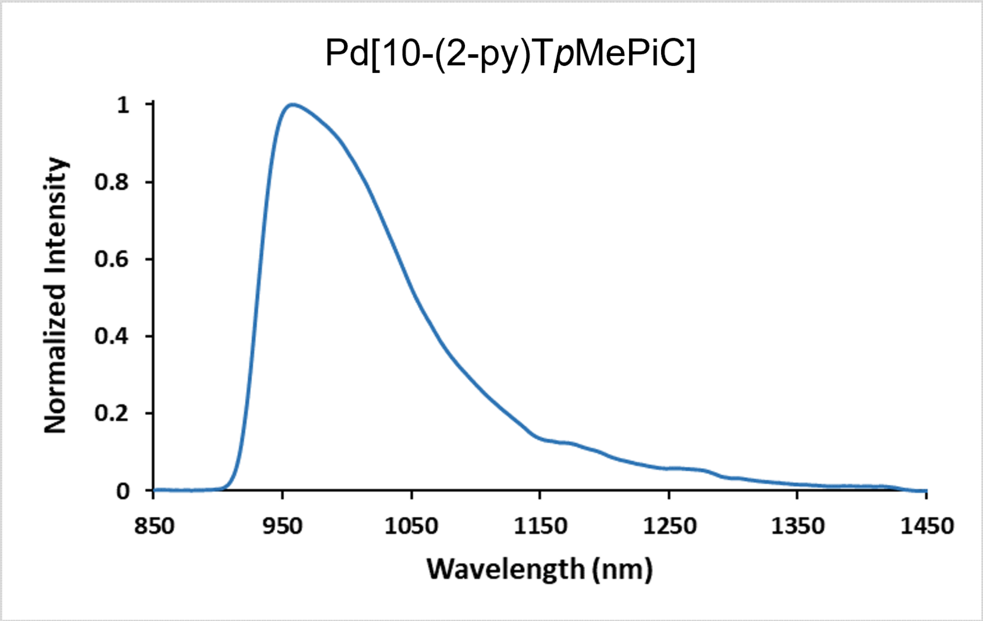

Figure S10: Phosphorescence of Pd[10-(2-py)T*p*MePiC] at 1.0 x 10^{-4} M in anoxic dichloromethane: 1 sec integration time, bandpass 26 nm, average of 5 scans.

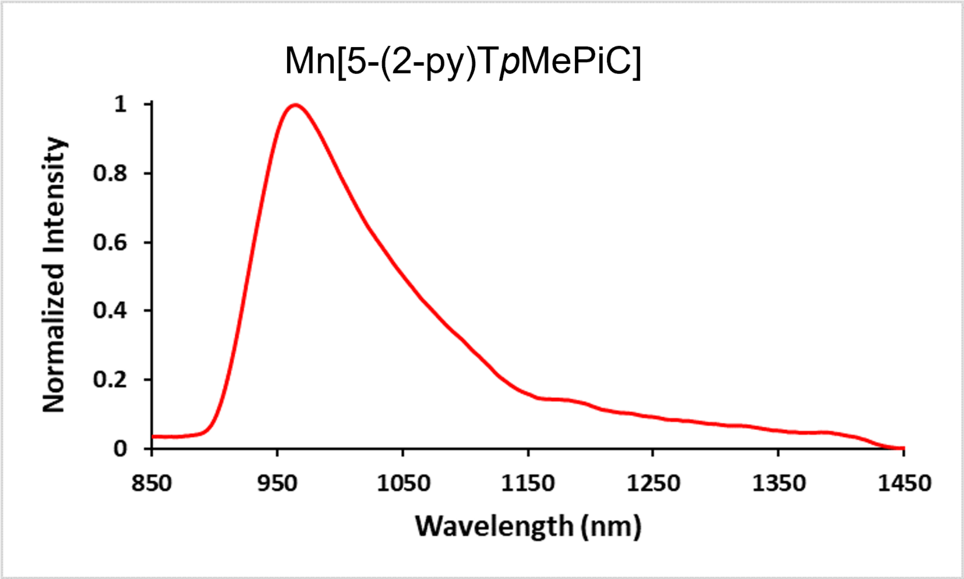

Figure S11: Phosphorescence of Mn[5-(2-py)T*p*MePiC] at 1.0 x 10^{-4} M in anoxic dichloromethane: 1 sec integration time, bandpass 50 nm, average of 5 scans.

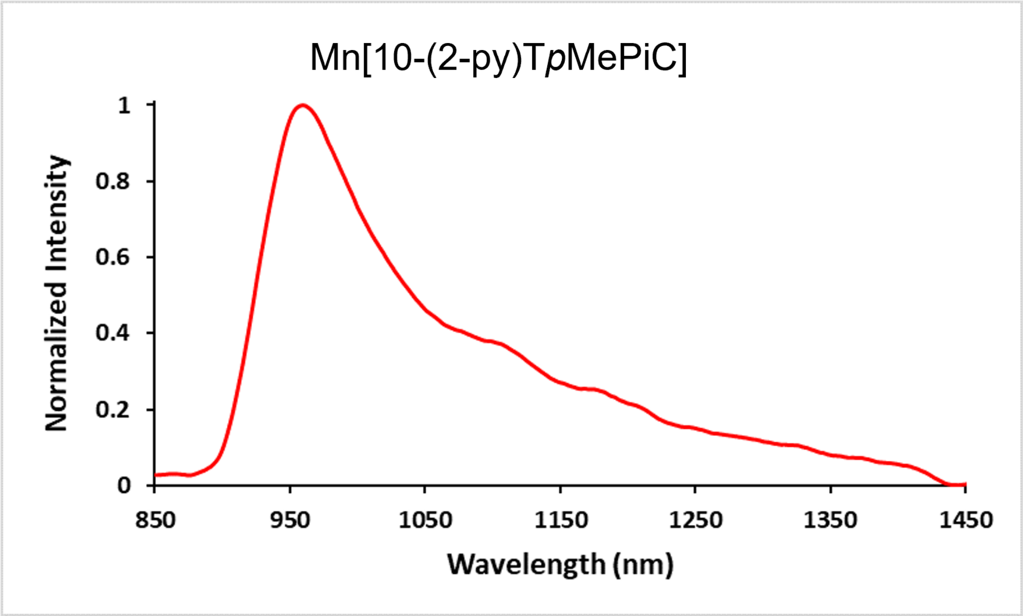

Figure S12: Phosphorescence of Mn[10-(2-py)T*p*MePiC] at 1.1 x 10^{-4} M in anoxic dichloromethane: 1 sec integration time, bandpass 50 nm, average of 5 scans.

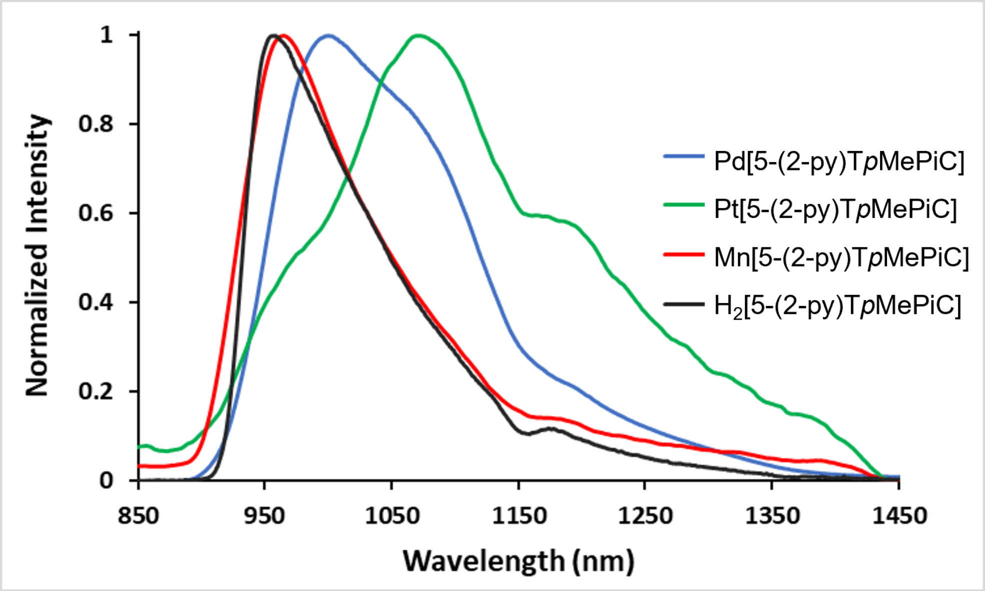

Figure S13: Comparison of isocorroles with 5-pyrrole appendage

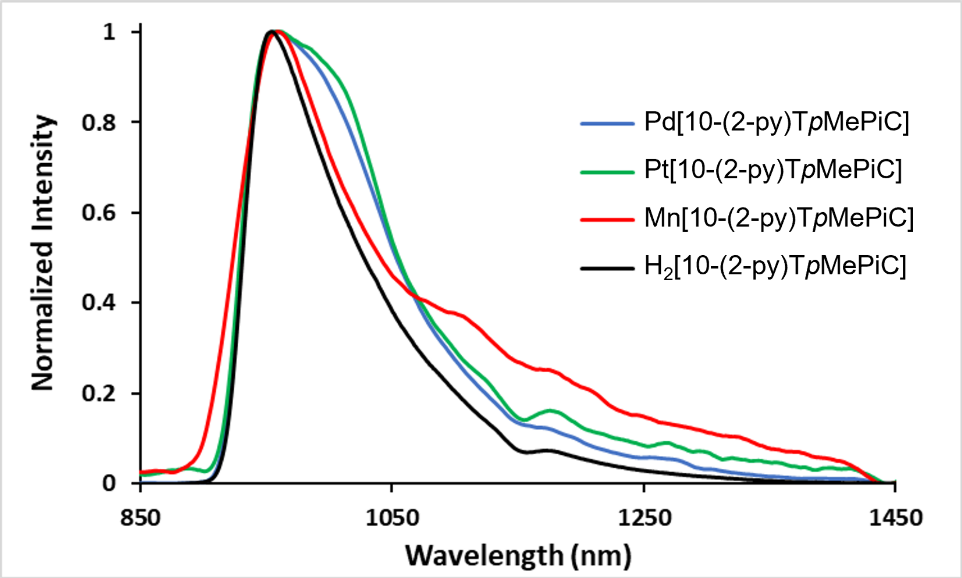

Figure S14: Comparison of isocorroles with 10-pyrrole appendage

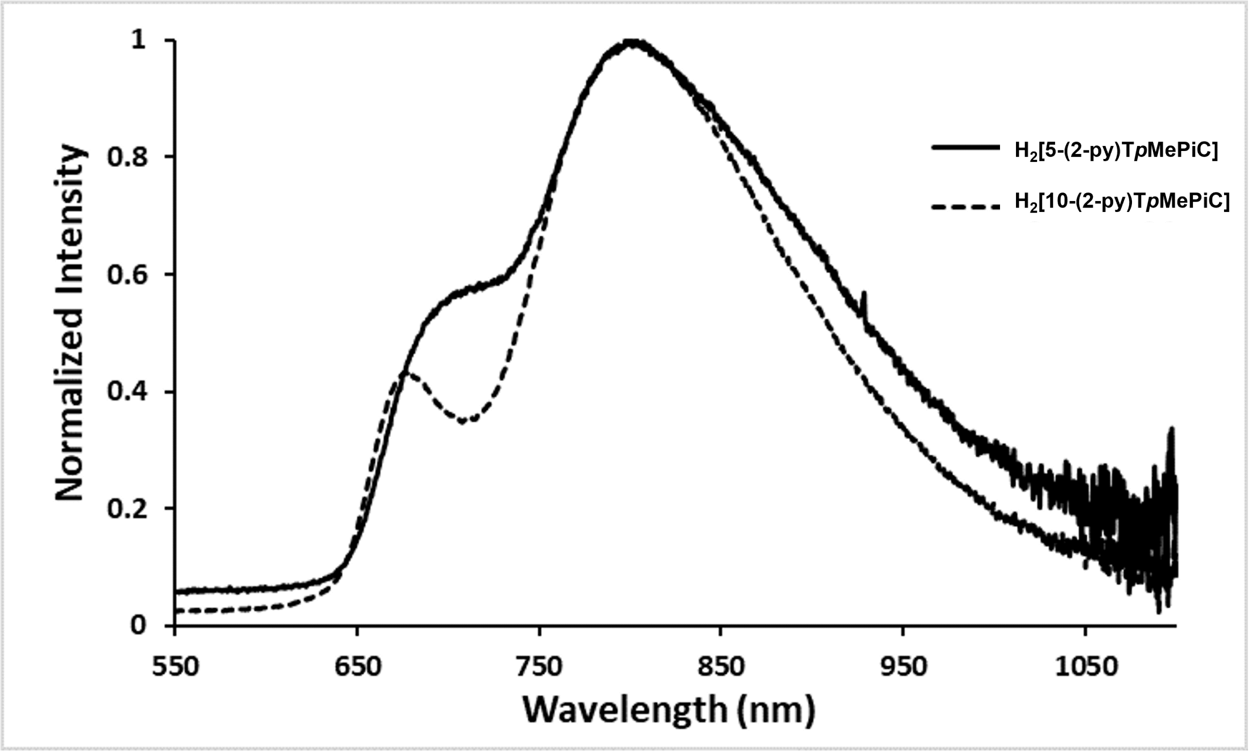

Figure S15: Visible-region emission (under 455 nm excitation) of free base isocorroles at 1.3 x 10^{-4} M in anoxic dichloromethane: 1 sec integration time, bandpass 20 nm.

C. Phosphorescence lifetime measurements

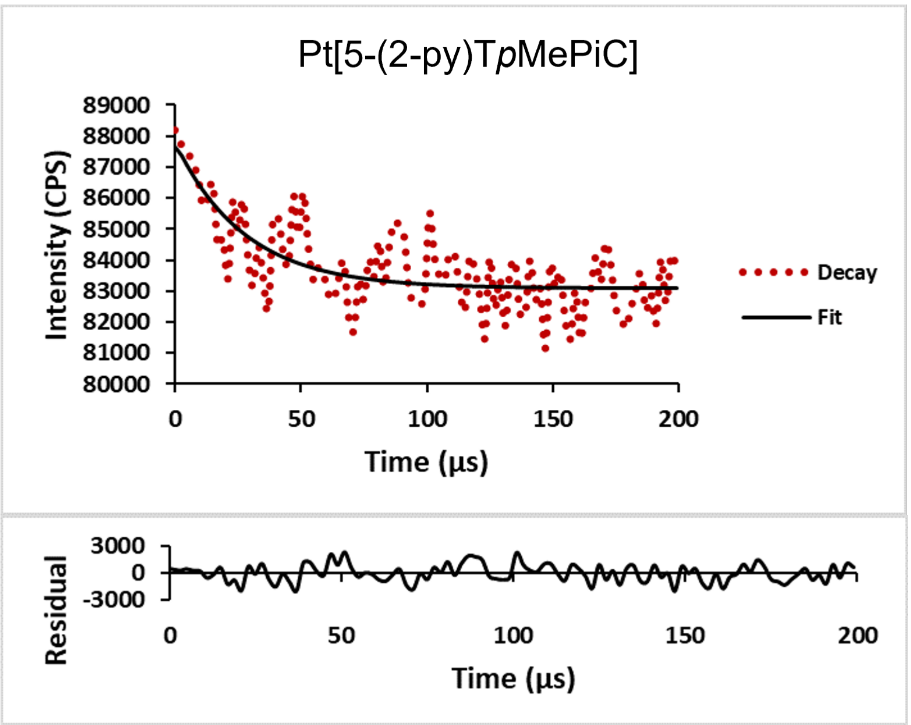

Figure S16: Lifetime of Pt[5-(2-py)T*p*MePiC] at 4.0 x 10^{-4} M. Average of 60 scans. Lifetime = 43 μs (1070 nm)

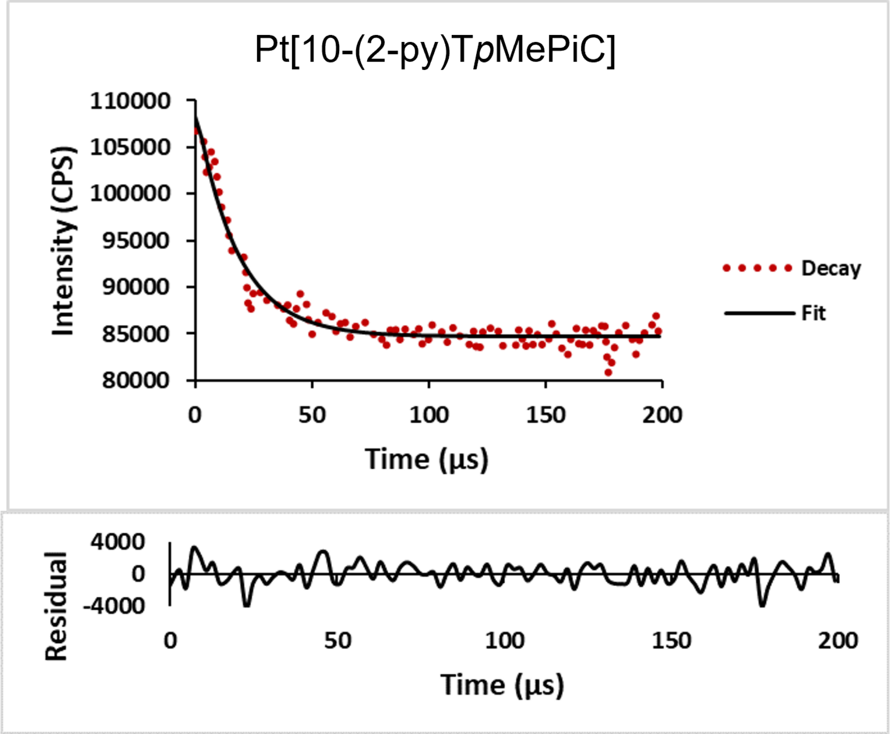

Figure S17: Lifetime of Pt[10-(2-py)T*p*MePiC] at 1.2 x 10^{-4} M. Average of 60 scans. Lifetime = 25 μs (965 nm)

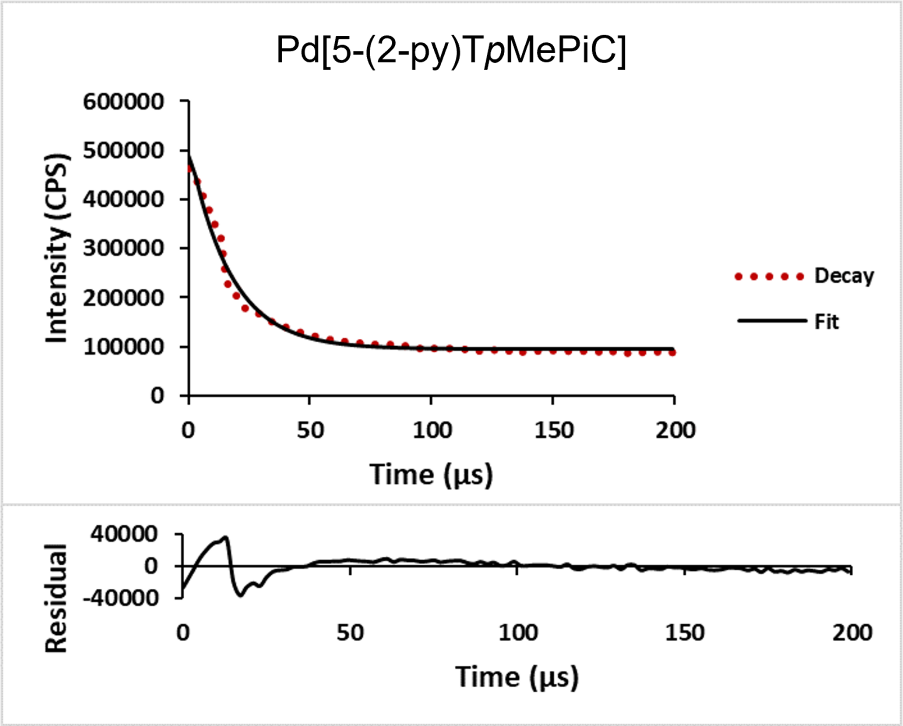

Figure S18: Lifetime of Pd[5-(2-py)T*p*MePiC] at 4.1 x 10^{-6} M. Average of 30 scans. Lifetime = 26 μs (965 nm)

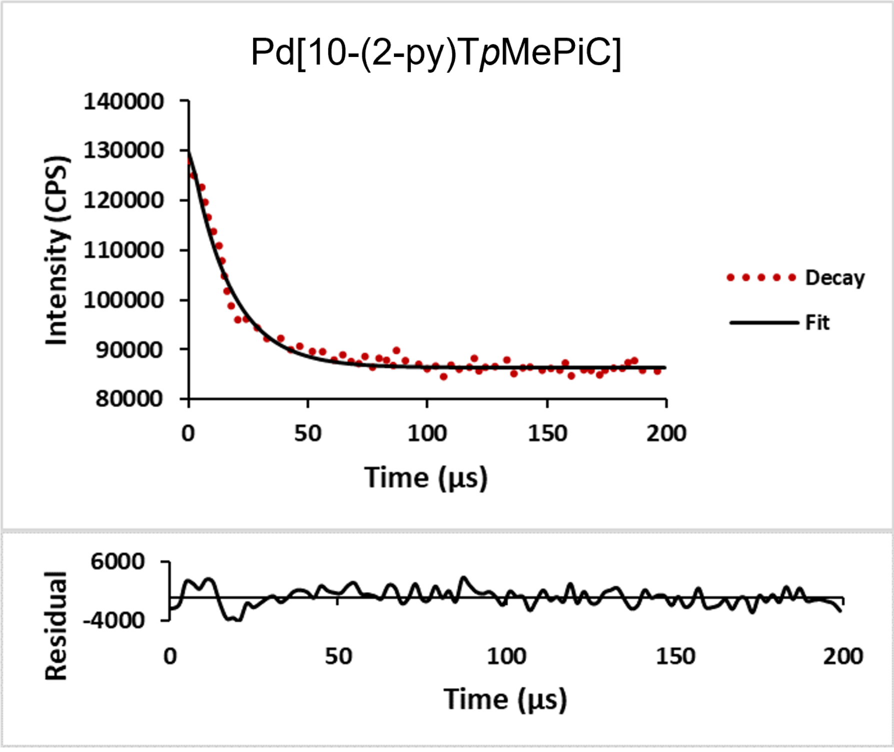

Figure S19: Lifetime of Pd[10-(2-py)T*p*MePiC] at 1.0 x 10^{-5} M. Average of 30 scans. Lifetime = 25 μs (965 nm)

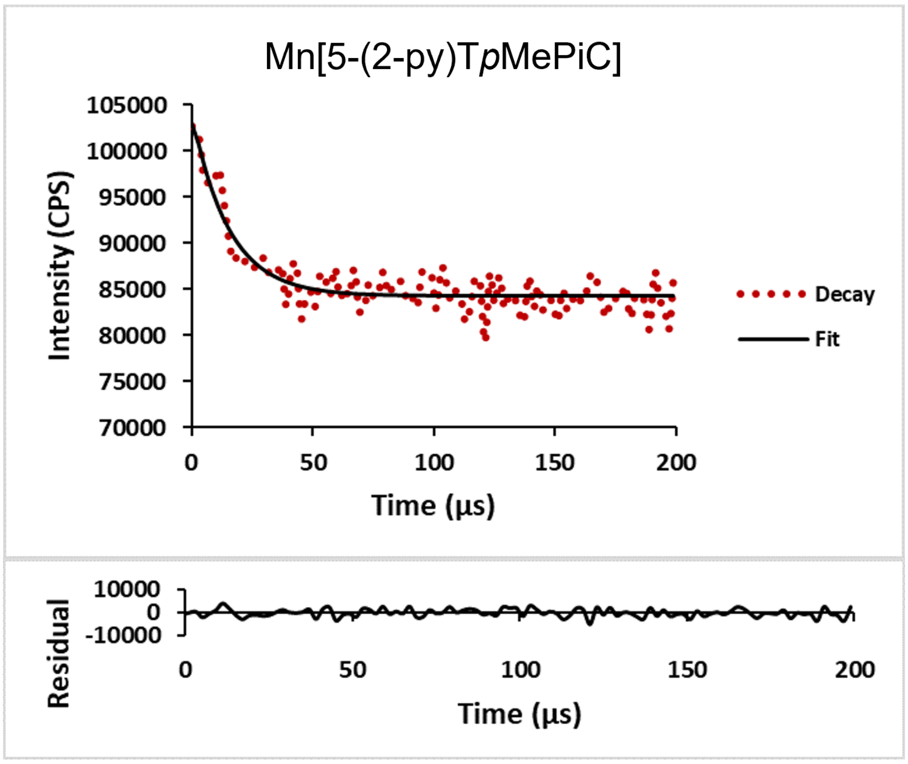

Figure S20: Lifetime of Mn[5-(2-py)T*p*MePiC] at 1.0 x 10^{-4} M. Average of 20 scans. Lifetime = 23 μs (965 nm)

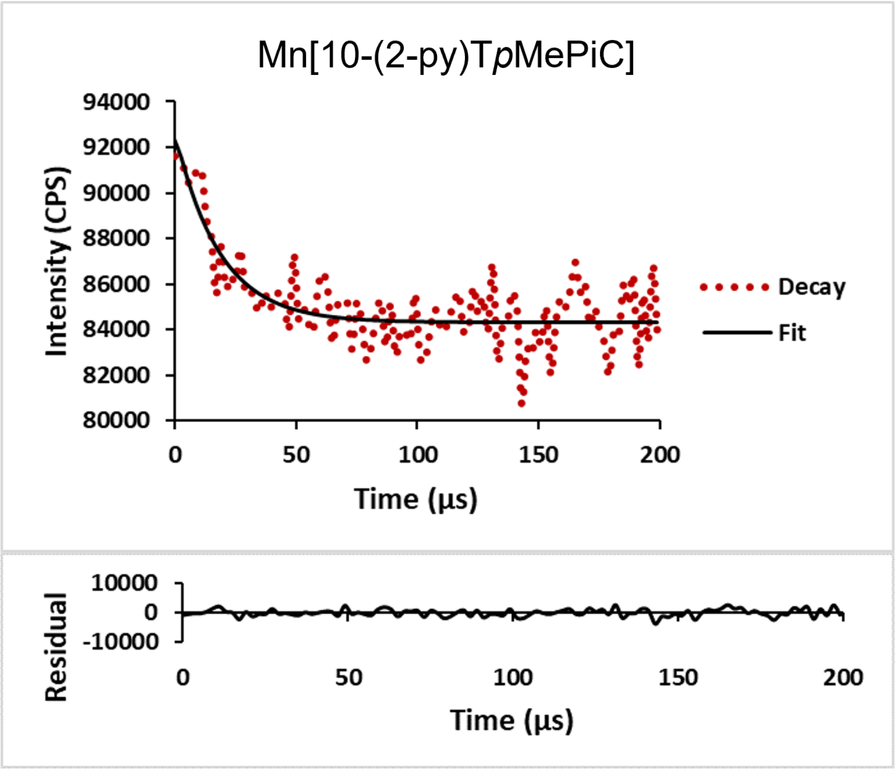

Figure S21: Lifetime of Mn[10-(2-py)T*p*MePiC] at 1.1 x 10^{-4} M. Average of 50 scans. Lifetime = 28 μs (965 nm)

D. Singlet oxygen sensitization measurements

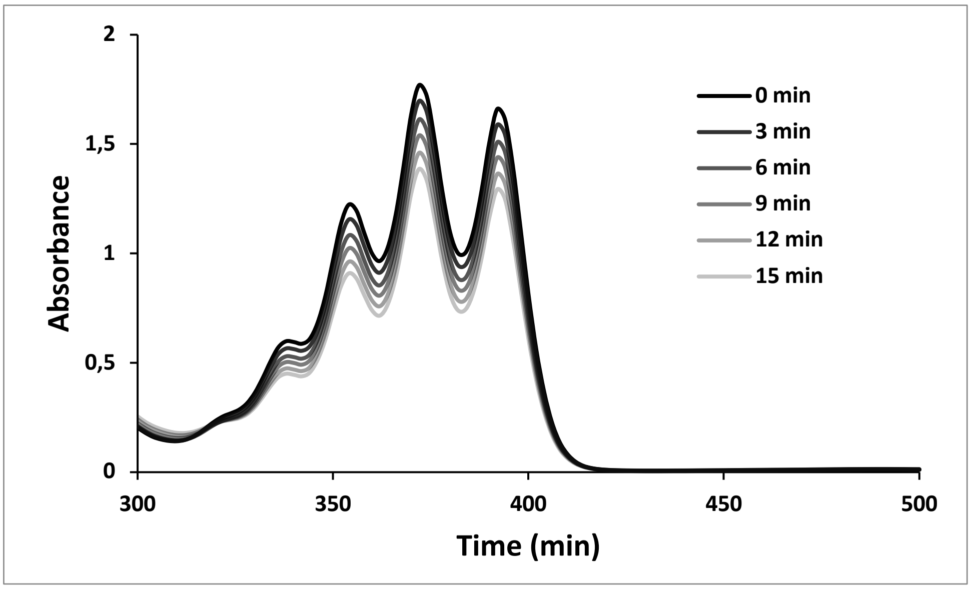

Figure S22: Absorbance spectra showing degradation of 9,10-diphenylanthracene (0.28 mM) in an air-saturated solution (9:1 EtOH/THF) in the presence of methylene blue (5 µM) upon irradiation with 405 nm LED.

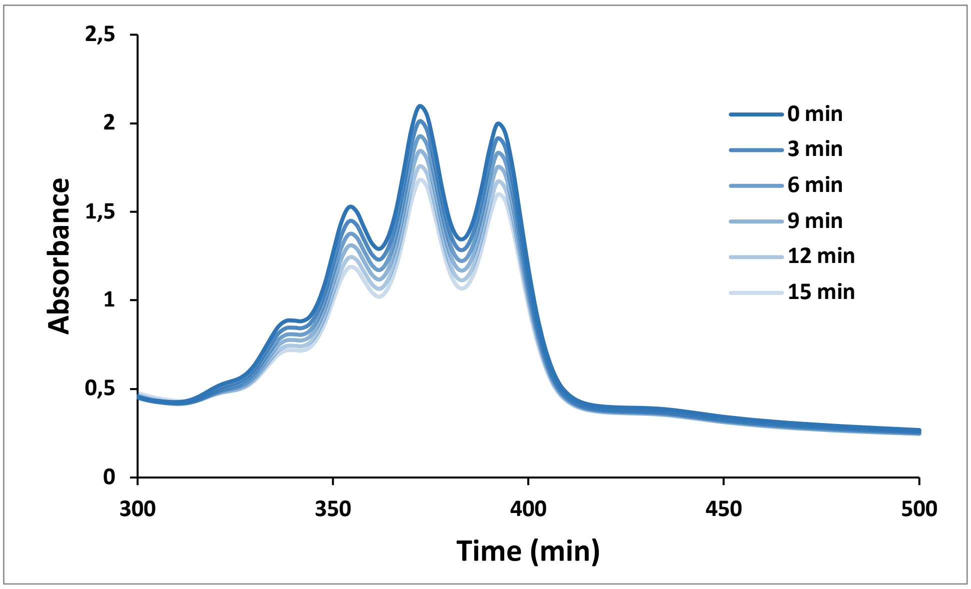

Figure S23: Absorbance spectra showing degradation of 9,10-diphenylanthracene (0.28 mM) in an air-saturated solution (9:1 EtOH/THF) in the presence of Pt[10-(2-py)T*p*MePiC] (5 µM) upon irradiation with 405 nm LED.

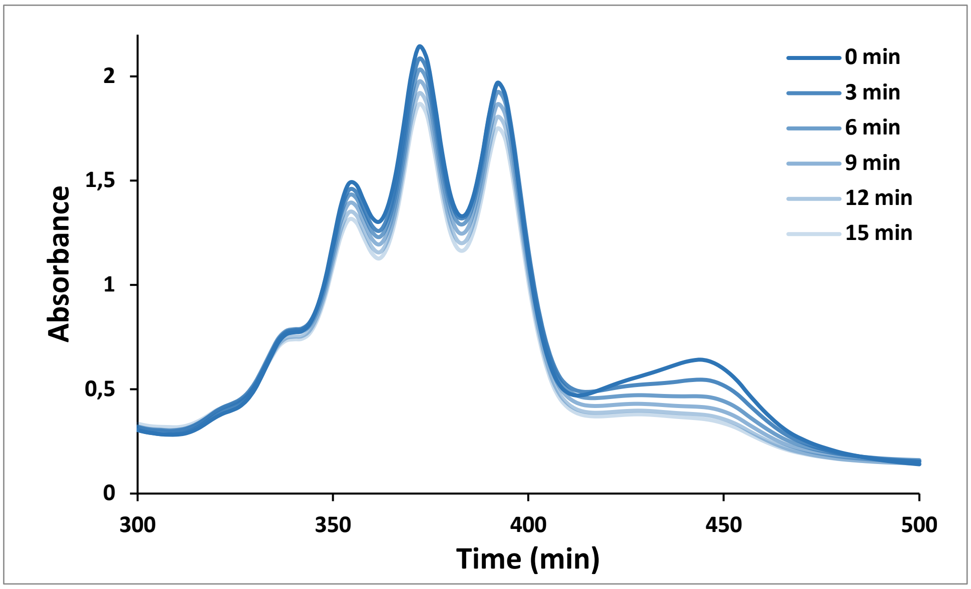

Figure S24: Absorbance spectra showing degradation of 9,10-diphenylanthracene (0.28 mM) in an air-saturated solution (9:1 EtOH/THF) in the presence of Pd[5-(2-py)T*p*MePiC] (5 µM) upon irradiation with 405 nm LED.

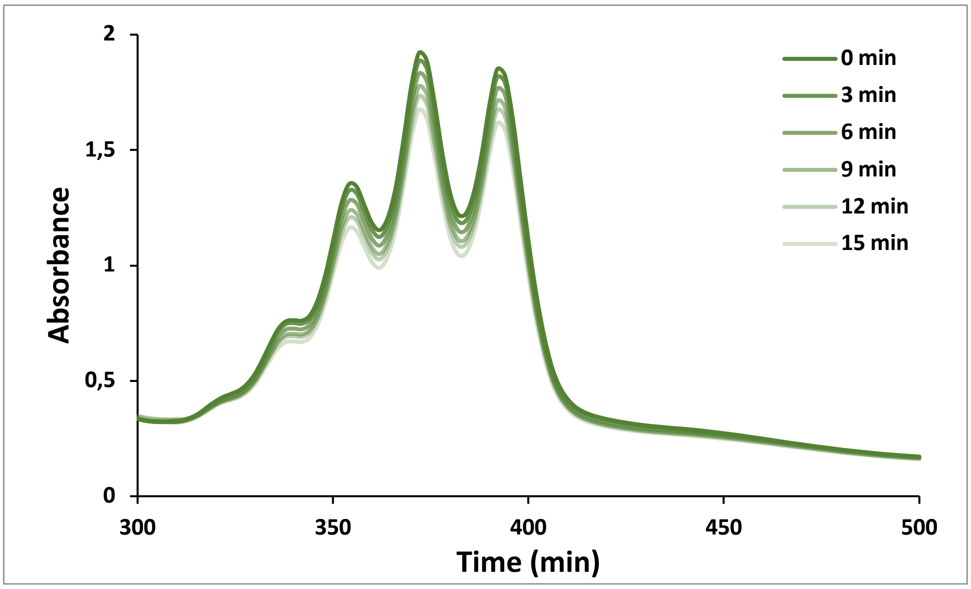

Figure S25: Absorbance spectra showing degradation of 9,10-diphenylanthracene (0.28 mM) in an air-saturated solution (9:1 EtOH/THF) in the presence of Pt[10-(2-py)T*p*MePiC] (5 µM) upon irradiation with 405 nm LED.

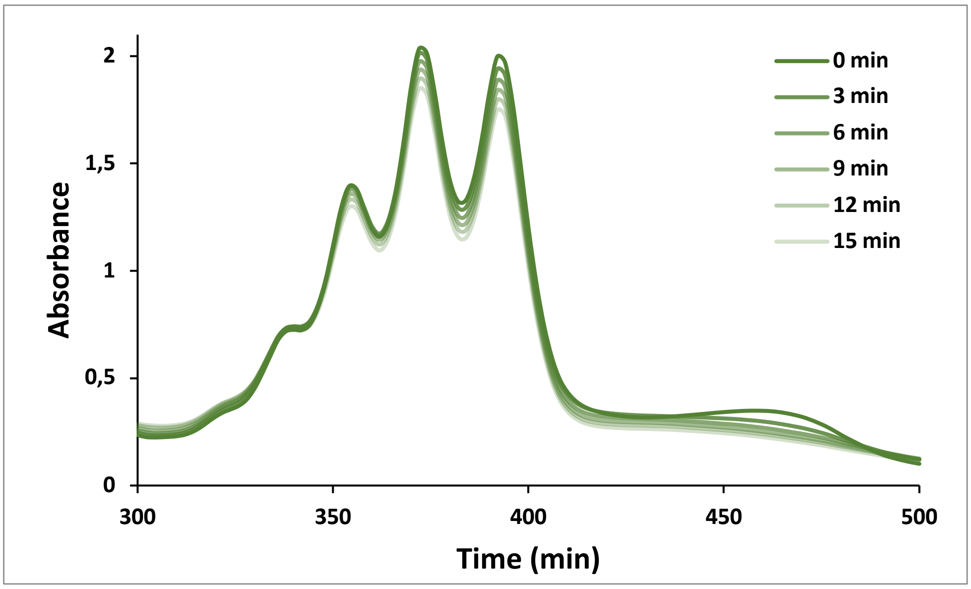

Figure S26: Absorbance spectra showing degradation of 9,10-diphenylanthracene (0.28 mM) in an air-saturated solution (9:1 EtOH/THF) in the presence of Pt[5-(2-py)T*p*MePiC] (5 µM) upon irradiation with 405 nm LED.

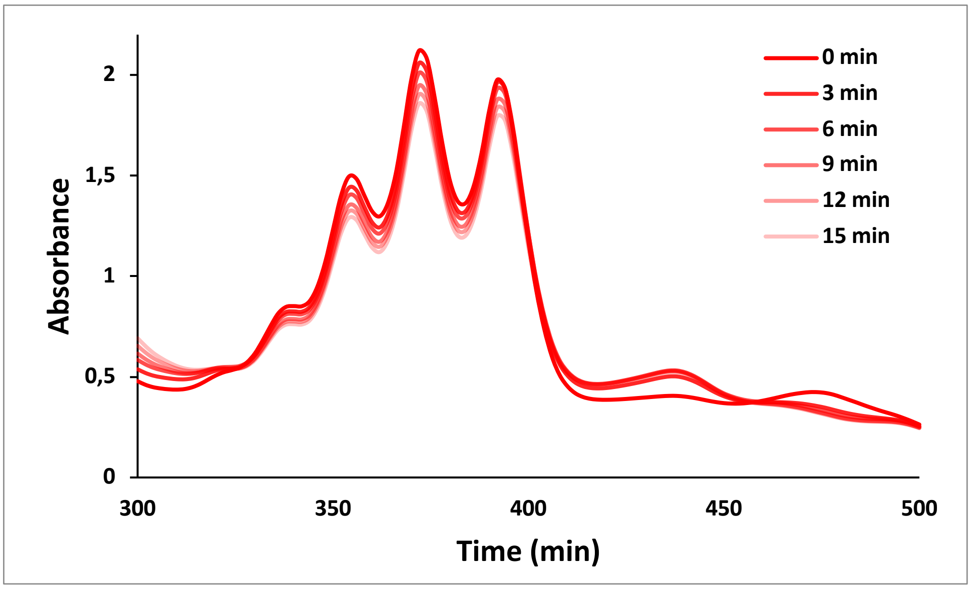

Figure S27: Absorbance spectra showing degradation of 9,10-diphenylanthracene (0.28 mM) in an air-saturated solution (9:1 EtOH/THF) in the presence of Mn[10-(2-py)T*p*MePiC] (5 µM) upon irradiation with 405 nm LED.

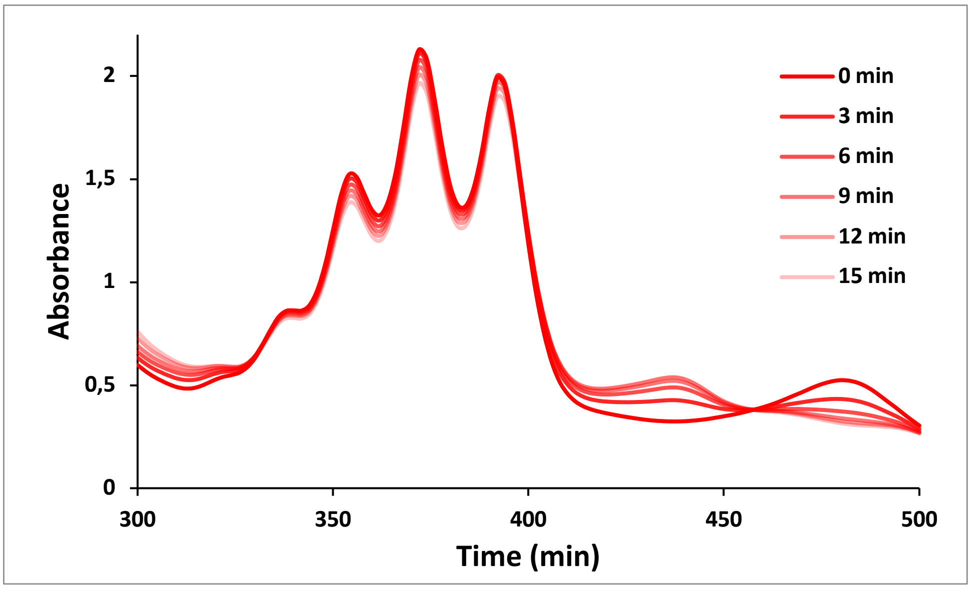

Figure S28: Absorbance spectra showing degradation of 9,10-diphenylanthracene (0.28 mM) in an air-saturated solution (9:1 EtOH/THF) in the presence of Mn[5-(2-py)T*p*MePiC] (5 μM) upon irradiation with 405 nm LED.
